# Supplementary material for: A standardized genome architecture for bacterial synthetic biology (SEGA)
Source: Nat Commun. 2021 Oct 7;12:5876. doi: 10.1038/s41467-021-26155-5 (PMC8497626; doi:10.1038/s41467-021-26155-5)
Supplement: Supplementary file 1 — Supplementary Information [file 41467_2021_26155_MOESM1_ESM.pdf]

Supporting information for:

A standardized genome architecture for bacterial synthetic biology (SEGA)

Carolyn N. Bayer[1†], Maja Rennig[1†]*, Anja K. Ehrmann[1†] and Morten H.H. Nørholm[1]*

[1]Novo Nordisk Foundation Center for Biosustainability, Technical University of Denmark, Kgs. Lyngby, Denmark

[†] These authors contributed equally

* Correspondence to MR (rennig@biosustain.dtu.dk) or MHHN (morno@biosustain.dtu.dk)

Supplementary Figure 1.

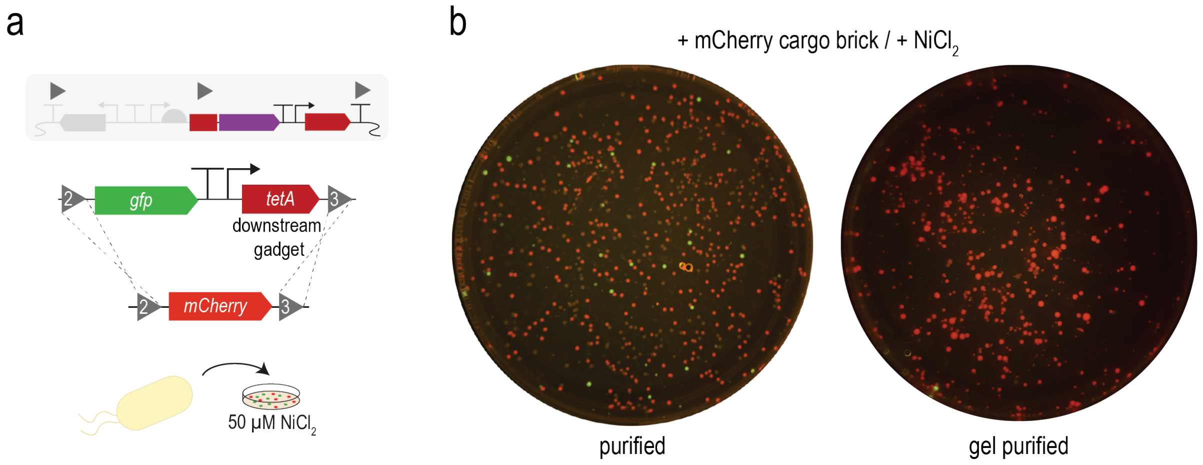

Supplementary Figure 1. mCherry integration efficiencies. (a) Schematic of the integration of a SEGA cargo brick (mCherry) utilizing the *tetA* gadget. **(b)** Plates from integration of a spin column-purified (left) and a gel purified (right) mCherry cargo brick. Positive recombinants were selected on $NiCl_2$.

Supplementary Figure 2.

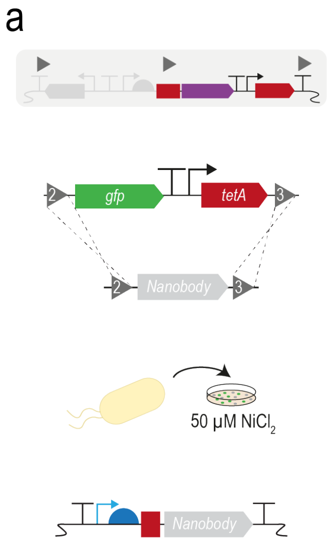

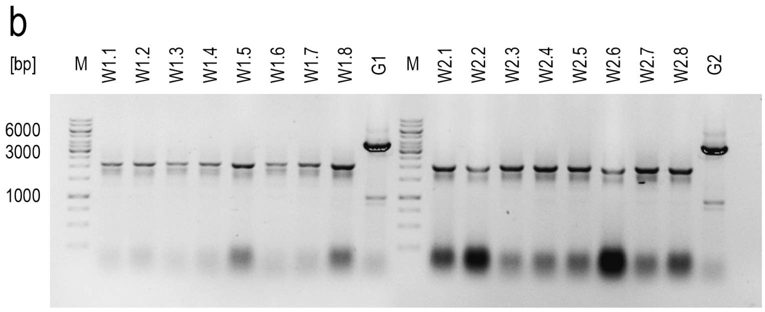

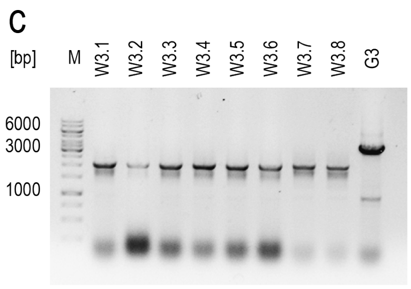

d

replicate	white	green	%white
Rep 1	1880	117	94.1%
Rep 2	1520	195	88.6%
Rep 3	1036	248	80.7%

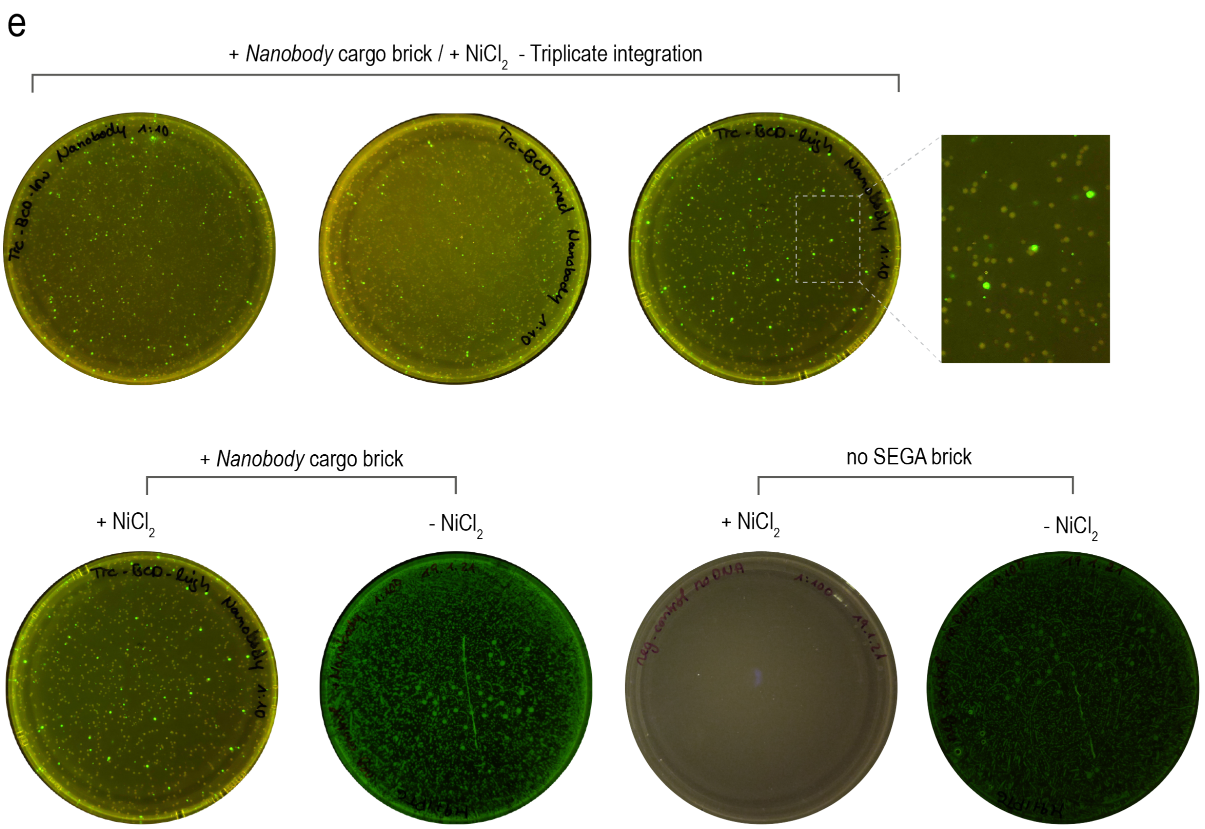

Supplementary Figure 2. Integration of a Nanobody cargo brick utilizing green-white screening.

(a) Schematic of the integration of a SEGA cargo brick utilizing green-white screening, exemplified by the coding sequence for the Nanobody enhancer[1]. **(b), (c)** Colony PCR results from 3 independent integrations, screening 8 white and 1 green colony each. Size of the amplicon for the strating strain (Ptrc-BCD-*gfp-tetA*): 3761 bp; for the correct recombinants: 2136 bp. **(d)** Ratios of white and green colonies for 3 independent integrations. **(e)** Plates from 3 independent integrations of the Nanobody

cargo brick, visualizing green-white screening to select positive recombinants. The lower panel also includes the controls with and without $NiCl_2$ selection, and with and without the addition of the SEGA brick. Uncropped gel pictures are provided in the Source Data file.

Supplementary Figure 3.

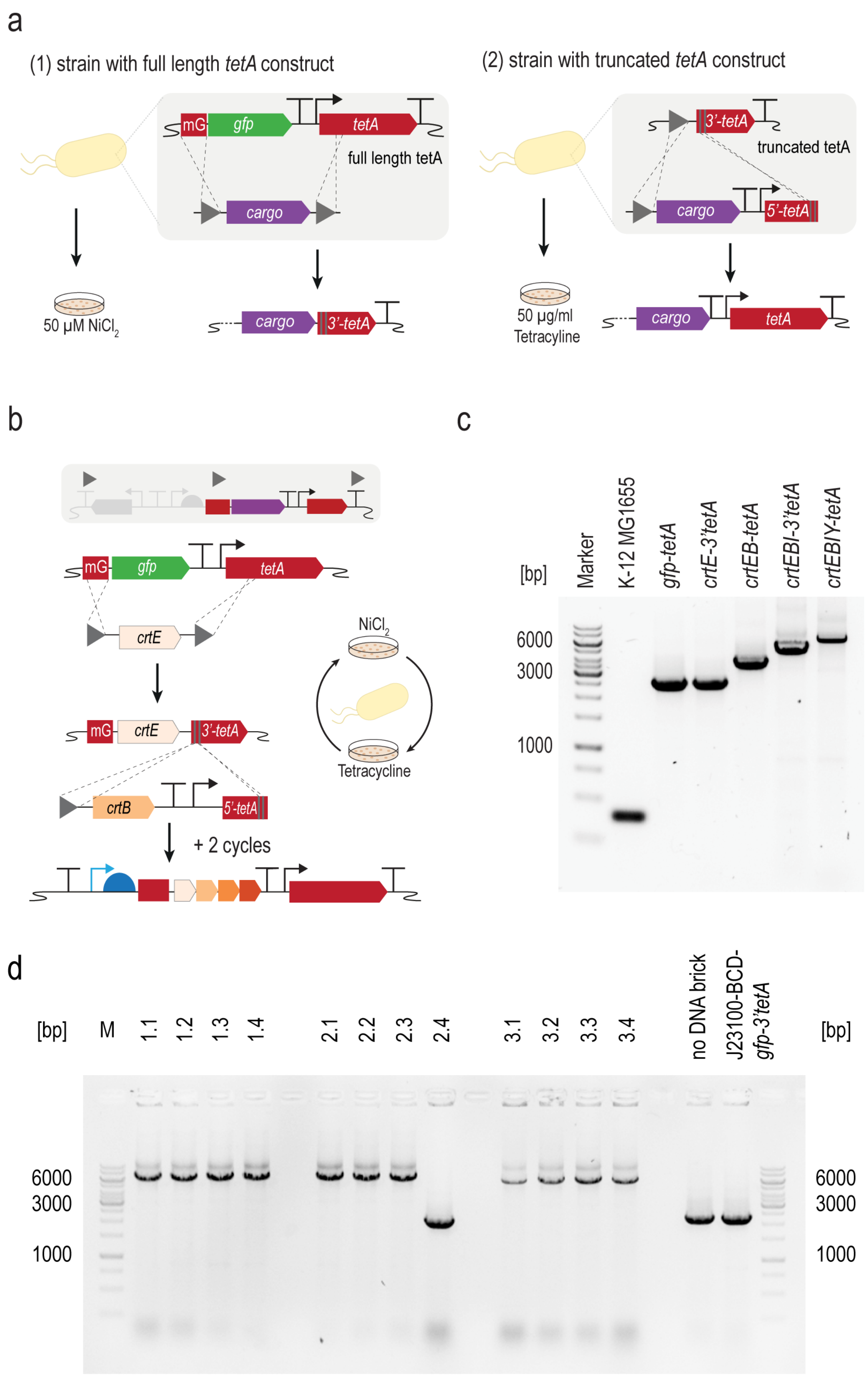

Supplementary Figure 3. Utilization of the truncated *tetA* gadget

(a) Schematic visualization of the application of the truncated *tetA* gadget. Truncation of *tetA* is enabled by counter-selection against functional *tetA* with 50 µM $NiCl_2$. The functional *tetA* can be reconstituted with a DNA fragment carrying the 5'*tetA* truncation. **(b)** The truncated *tetA* gaget enables cycling through multiple rounds of engineering within the SEGA landing pad. **(c)** Example of iterative genome engineering using the *tetA* gadget. The four genes of the *crtEBIY* operon are integrated sequentially as shown by colony PCR of the resulting strains. **(d)** Colony PCR results for four white/orange appearing colonies from three independent integrations of the *crtEBIY-5'tetA* cargo brick into the J23100-BCD-high-*gfp-3'tetA* strain, and negative controls. The size of the correct insert is 6376 bp. The agar plate corresponding to sample 3 in this figure is shown in Figure 2e. Uncropped gel pictures are provided in the Source Data file.

Supplementary Figure 4.

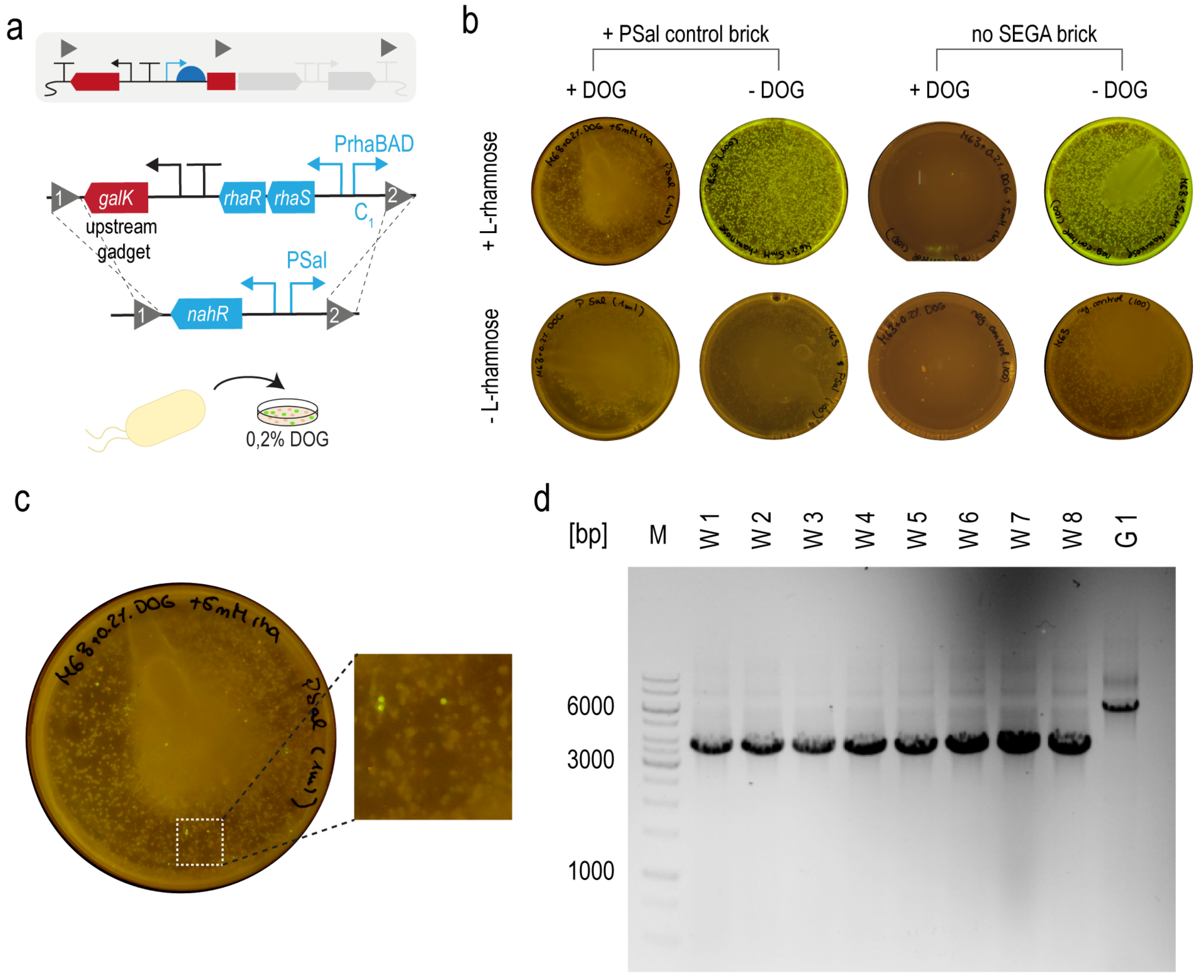

Supplementary Figure 4. Integration of a double stranded SEGA control brick and galK counter-selection

(a) Schematic visualization of the application of the *galK* gadget for integration of a PSal control brick. **(b)** Counter-selection against *galK* is achieved by addition of 0.2% 2-deoxy-galactose (DOG). Green-white screening is activated by addition of 5 mM L-rhamnose, which leads to green fluorescence of false positive colonies. **(c)** Zoomed view on an agar plate from (b) for green-white screening after exchange of the control elements through integration of the inducible PSal promoter. **(d)** Colony PCR of 8 white colonies (W1 – W8) and one green colony (G1) after the control element exchange utilizing the *galK* gadget. Size of the correct construct: 3726 bp; size of the fragment before recombination: 5845 bp. Uncropped gel pictures are provided in the Source Data file.

Supplementary Figure 5.

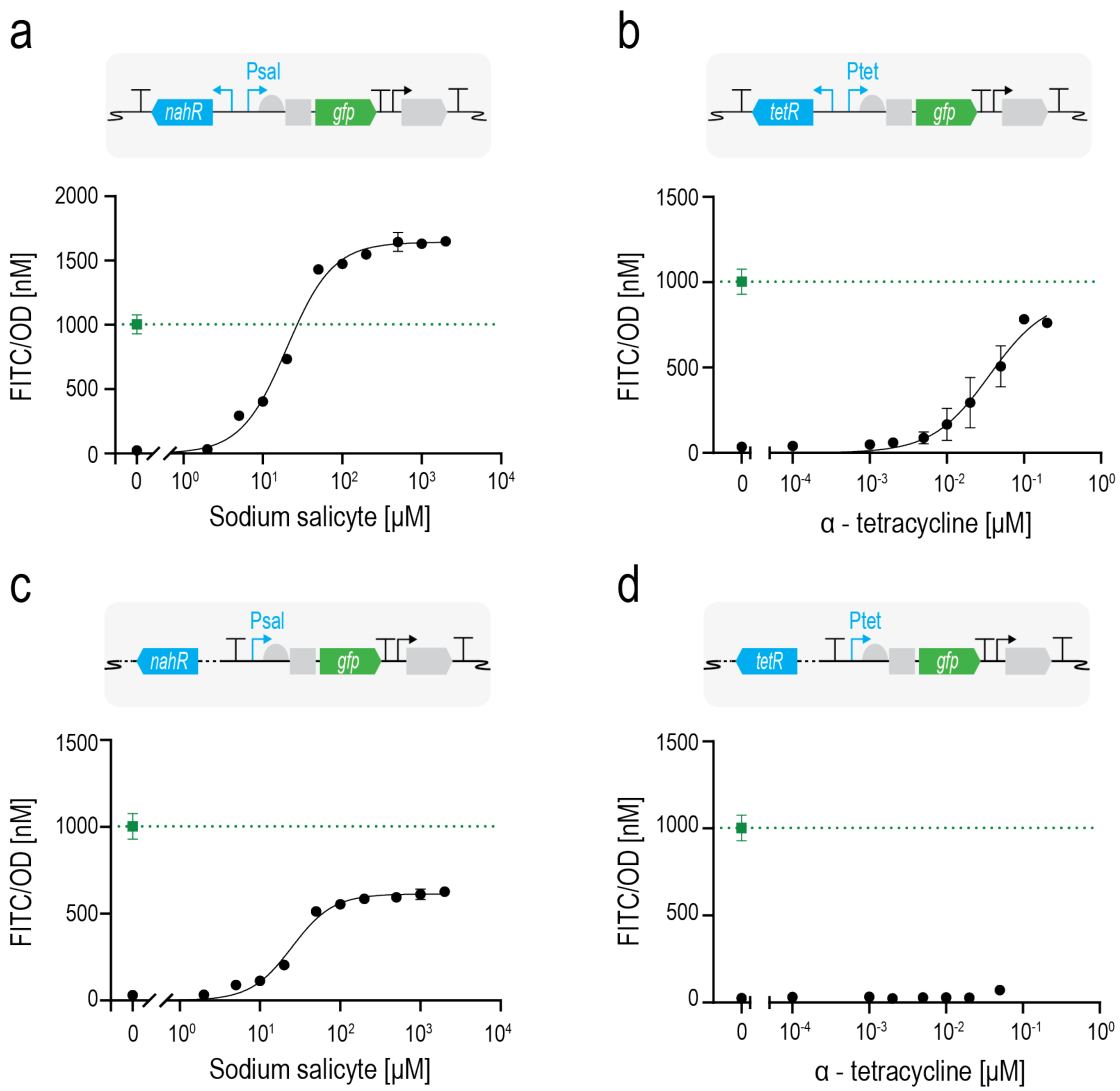

Supplementary Figure 5. GFP expression data with PSal and PTet as control element 1

The regulators NahR and TetR from the Marionette biosensor collection[2] were recloned with their respective target promoters in a divergent orientation and assembled as a SEGA landing pad to serve as C1 element, with C2: high and mGadget: BCD (biscistronic design). Response curves measured 4 hours after induction are given for **(a)** the PSal promoter and **(b)** the PTet promoter. **(c)**, **(d)** PSal and PTet landing pads were also constructed without their regulators and integrated into the *glmS-pstS* intergenic region in the "Marionette-Wild" strain (sAJM.1506[2]), which harbors an array of all regulators from the sensor collection in a separate genome location. In all panels, data points show the mean of three biological replicates and error bars denote the standard deviation. The expression strength of the constitutive promoter J23100 is shown as an internal reference (green line). Source data are provided in the Source data file.

Supplementary Figure 6.

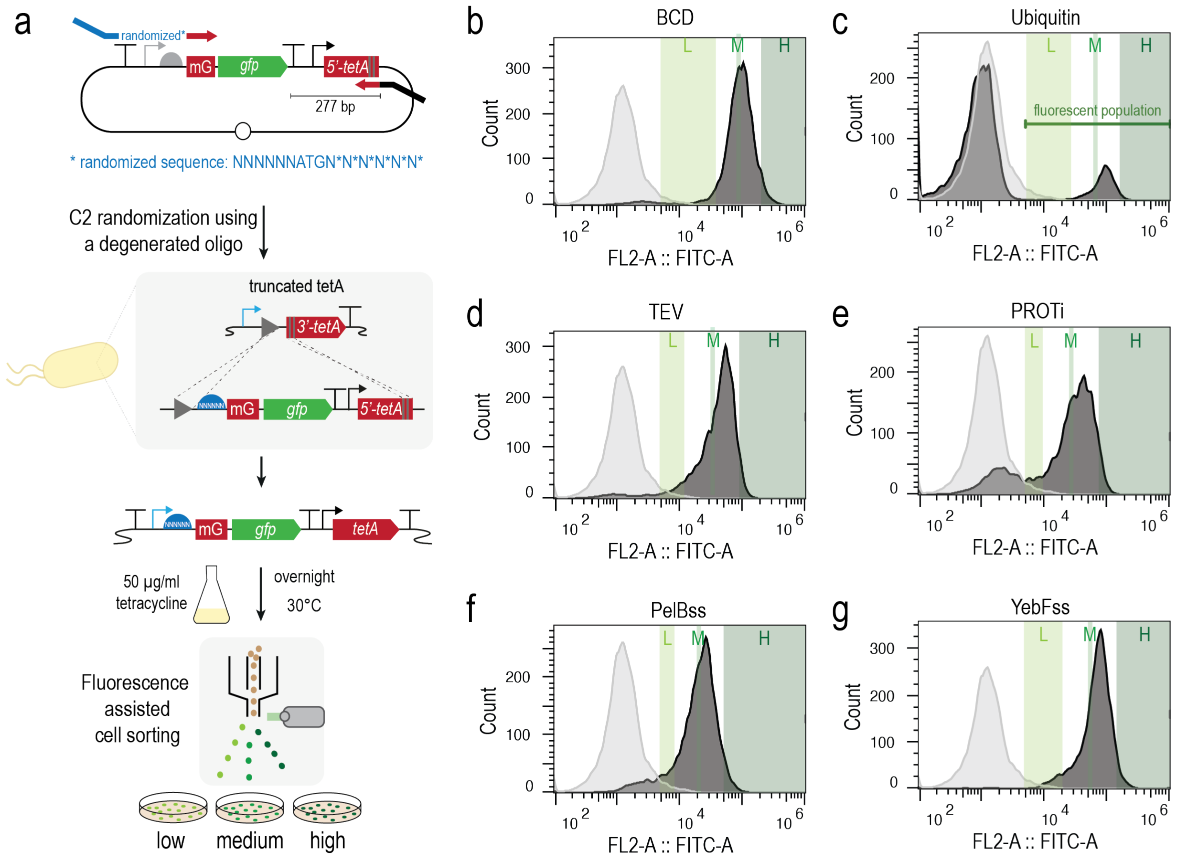

Supplementary Figure 6. FACS profiles of TIR libraries.

(a) Schematic of the workflow for generation of C2 libraries and selection of variants for low, medium and high translational strength. Panels **(b)** to **(g)** show the cell sorting gates applied to the C2 libraries for the selection of low (L), medium (M) and high (H) translational strength for the six different middle gadgets. Shown in light gray is the negative control (unengineered *E. coli* K12). Note to panel e): A C2 library was generated for the PROTi middle gadgets and variants for the translational strength were selected. However, the final C2-PROTi combinations were constructed by transferring the C2 sequences from the TEV strains, since these two gadgets share the first 21 bp. Flow cytometry dot plots used to identify the *E. coli* population of SEGA libraries based on forward scattered (FSC) and side scattered (SSC) light are shown in Supplementary Figure 13.

Supplementary Figure 7.

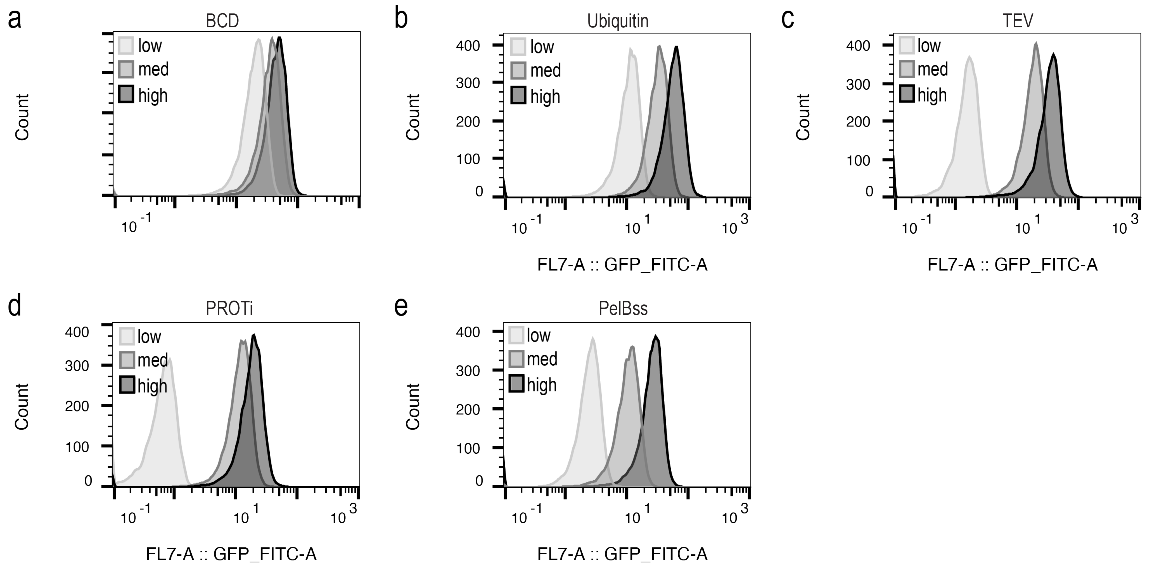

Supplementary Figure 7. Flow cytometry profile of selected low, medium and high expressing TIRs for BBa_J23100.

The preliminary *gfp* cargo was produced with a low, medium and high expressing TIR for the middle gadgets BCD **(a)**, Ubiquitin **(b)**, TEV **(c)**, PROTi **(d)** and PelBss **(e)**.

Supplementary Figure 8.

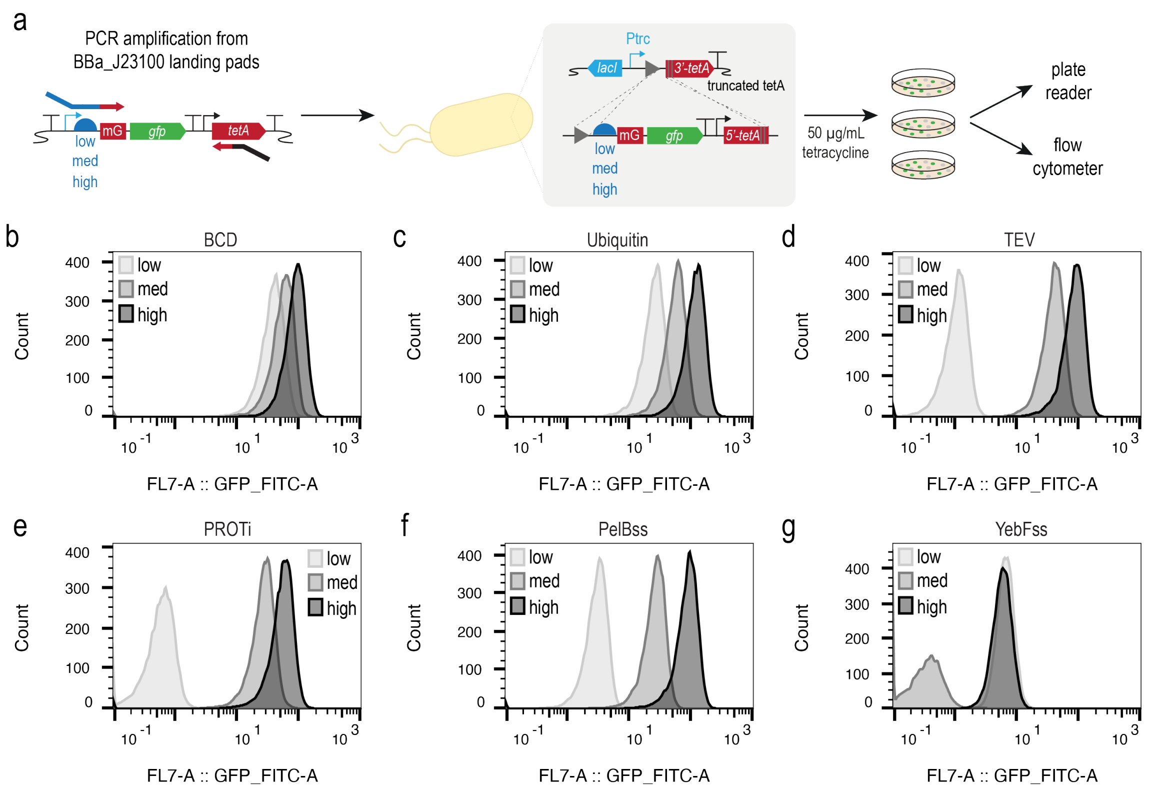

Supplementary Figure 8. Flow cytometry profile of selected low, medium and high expressing TIRs for Ptrc.

(a) SEGA landing pads containing the BBa_J23100 promoter were amplified by PCR and integrated into SEGA landing pads containing the *trc* promoter and the 5'-*tetA* gadget. Successful integrants were selected on tetracycline and performance of the prelimary *gfp* cargo analyzed by in a plate reader and by flow cytometry. The preliminary *gfp* cargo was produced with a low, medium and high expressing TIR for the middle gadgets BCD **(b)**, Ubiquitin **(c)**, TEV **(d)**, PROTi **(e)**, PelBss **(f)** and YebF **(g)**.

Supplementary Figure 9.

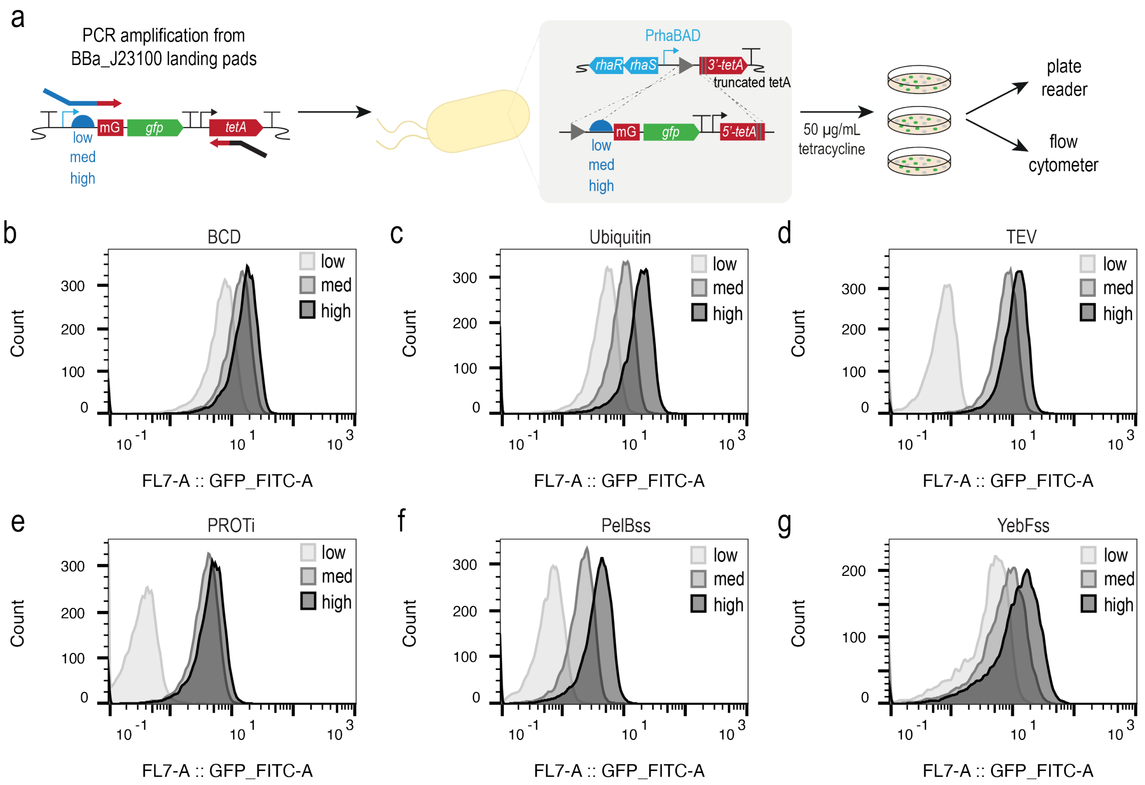

Supplementary Figure 9. Flow cytometry profile of selected low, medium and high expressing TIRs for PrhaBAD.

(a) SEGA landing pads containing the BBa_J23100 promoter were amplified by PCR and integrated into SEGA landing pads containing the *rhaBAD* promoter and the 5'-*tetA* gadget. Successful integrants were selected on tetracycline and performance of the prelimary *gfp* cargo analyzed by in a plate reader and by flow cytometry. The preliminary *gfp* cargo was produced with a low, medium and high expressing TIR for the middle gadgets BCD **(b)**, Ubiquitin **(c)**, TEV **(d)**, PROTi **(e)**, PelBss **(f)** and YebF **(g)**.

Supplementary Figure 10.

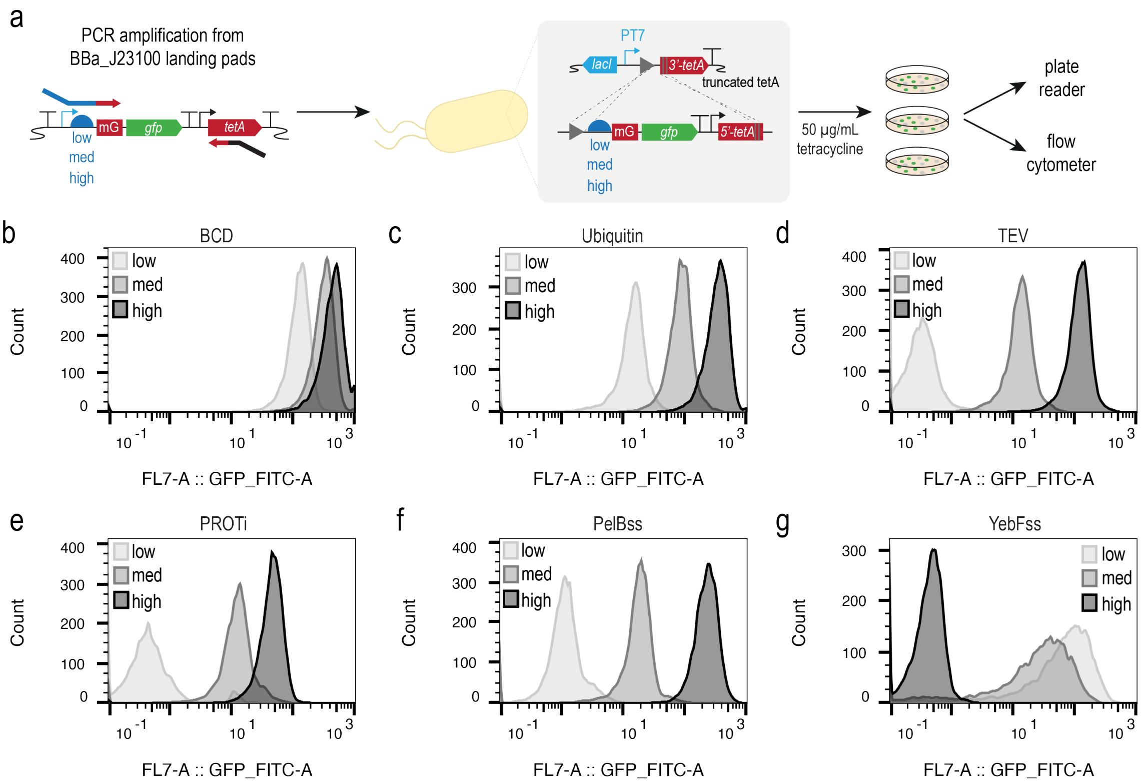

Supplementary Figure 10. Flow cytometry profile of selected low, medium and high expressing TIRs for PT7.

(a) SEGA landing pads containing the BBa_J23100 promoter were amplified by PCR and integrated into SEGA landing pads containing the T7 promoter and the 5'-*tetA* gadget. Successful integrants were selected on tetracycline and performance of the prelimary *gfp* cargo analyzed by in a plate reader and by flow cytometry. The preliminary *gfp* cargo was produced with a low, medium and high expressing TIR for the middle gadgets BCD **(b)**, Ubiquitin **(c)**, TEV **(d)**, PROTi **(e)**, PelBss **(f)** and YebF **(g)**.

Supplementary Figure 11.

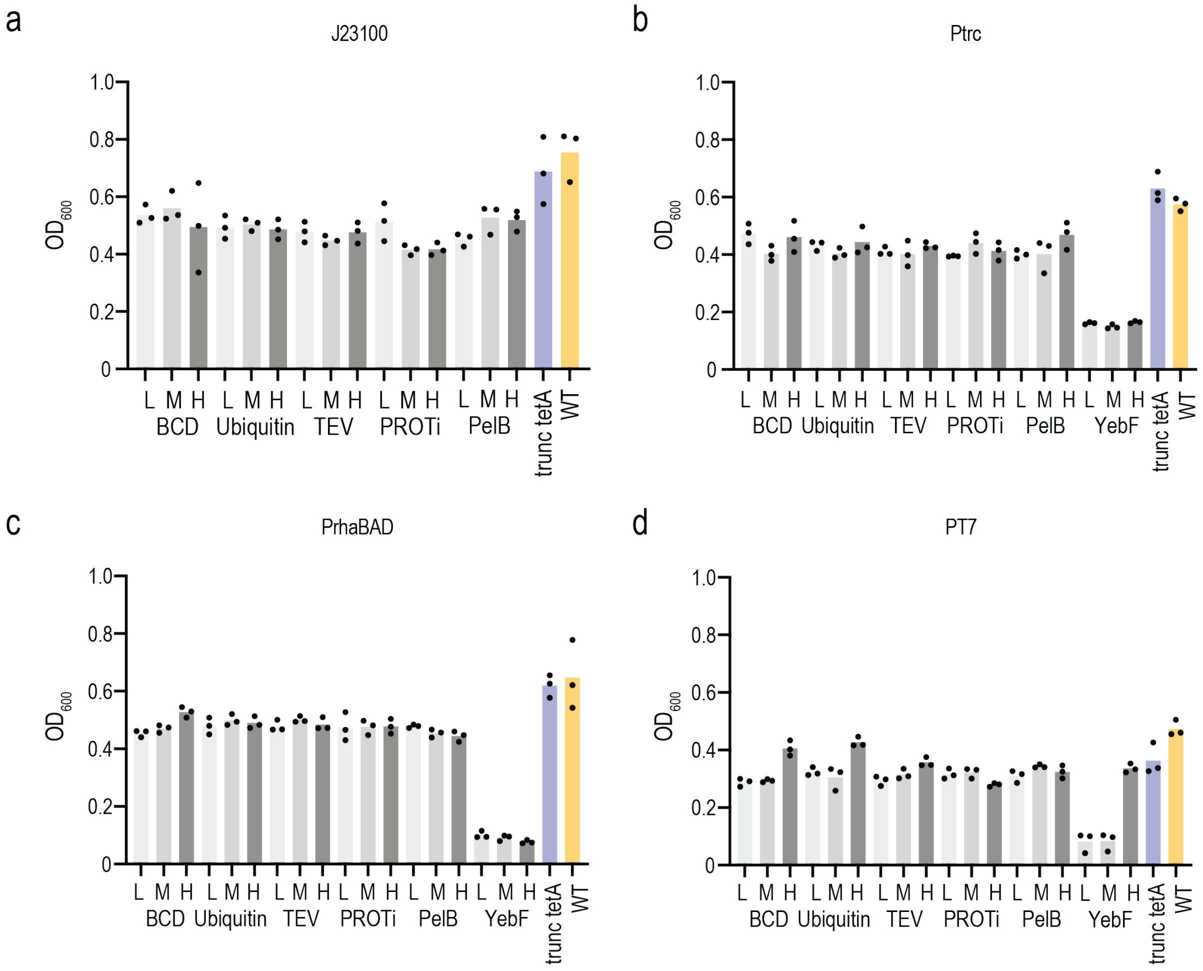

Supplementary Figure 11. Growth of SEGA strains.

Cells were grown in LB liquid medium and OD_{630} values were assessed after 7 hours of growth using a microtiter plate reader. Wildtype *E. coli* MG1655 (yellow) was included as well as the truncated tetA stain harboring the corresponding promoter (purple). The data corresponds to the GFP/OD measurements shown in Figure 3e and Figure 3h. The bars represent the mean of three biological triplicates. The datapoints of the three individual replicates are shown. Source data are provided in the Source Data file.

Supplementary Figure 12.

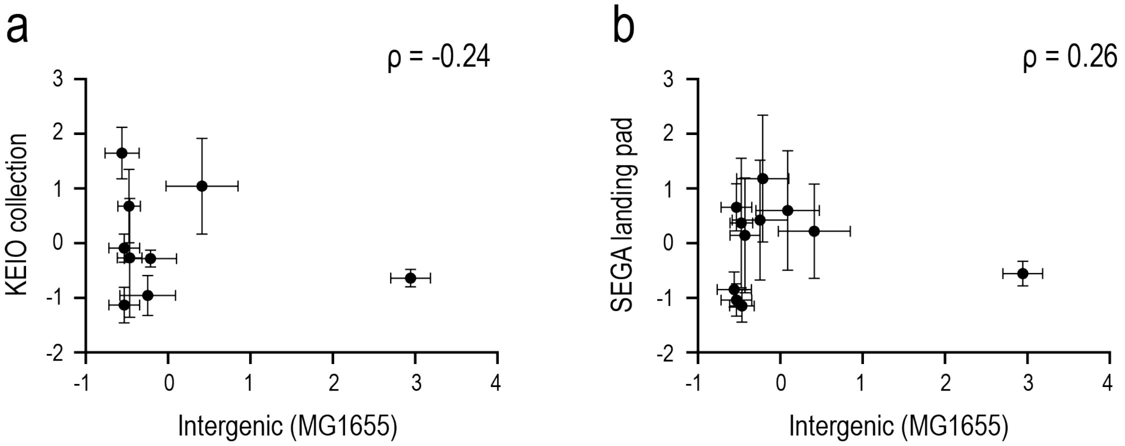

Supplementary Figure 12. Correlation of integration efficiencies for 11 genomic loci between different integration workflows.

Integration efficiency was compared between MG1655 intergenic integrations and integrations into the KEIO collection **(a)** and MG1655 intergenic integrations and integrations into the SEGA landing pad **(b)**. Counts of colony forming units were normalized by substracting the mean and deviding by the standard deviation of a respective replicate of the integration experiment. Each data point represents the integration into a particular genomic locus as depicted in Figure 5 in the main manuscript. Error bars show standard deviations across three replicates and the measure of center represents the mean. Spearman's rank correlation coefficient (ρ) was calculated using the spearmanr function from the scipy.stats package[3]. Source data are provided in the Source Data file.

Supplementary Figure 13.

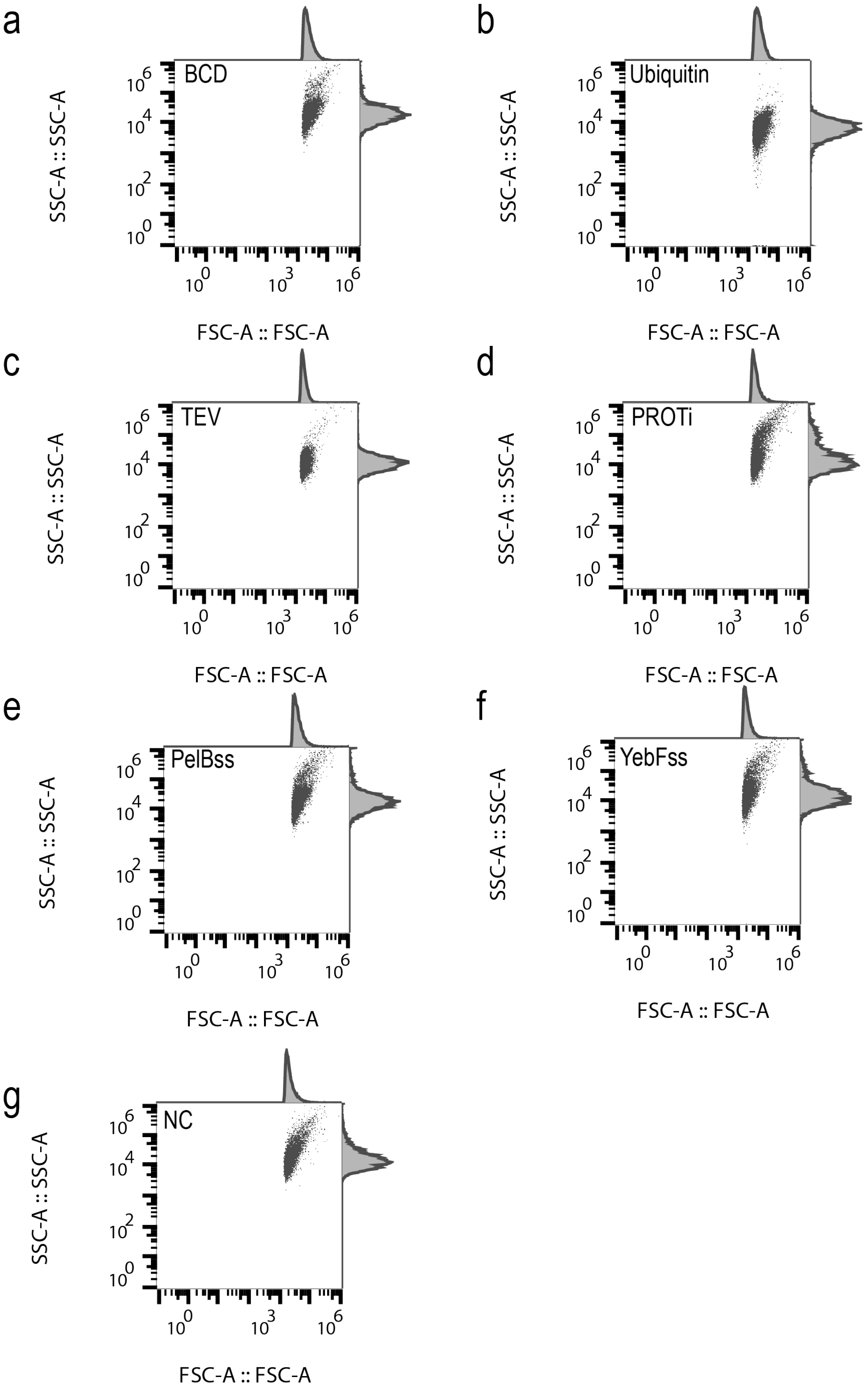

Supplementary Figure 13. Flow cytometry dot plots used to identify the *E. coli* population based on forward scattered (FSC) and side scattered (SSC) light.

The experimentaly threshold was set to 1.5% > FSC. No further gating strategy was applied and therefore no cells excluded from the experiment. Data are shown for libraries harbouring the middle gadgets BCD **(a)**, Ubiquitin **(b)**, TEV **(c)**, PROTi **(d)**, PelBss **(e)**, YebFss **(f)** and the BBa_J23100 promoter. Panel **(g)** depicts the negative control (NC). Raw data can be found on FlowRepository.org under the Repository ID FR-FCM-Z43R.

Supplementary Figure 14.

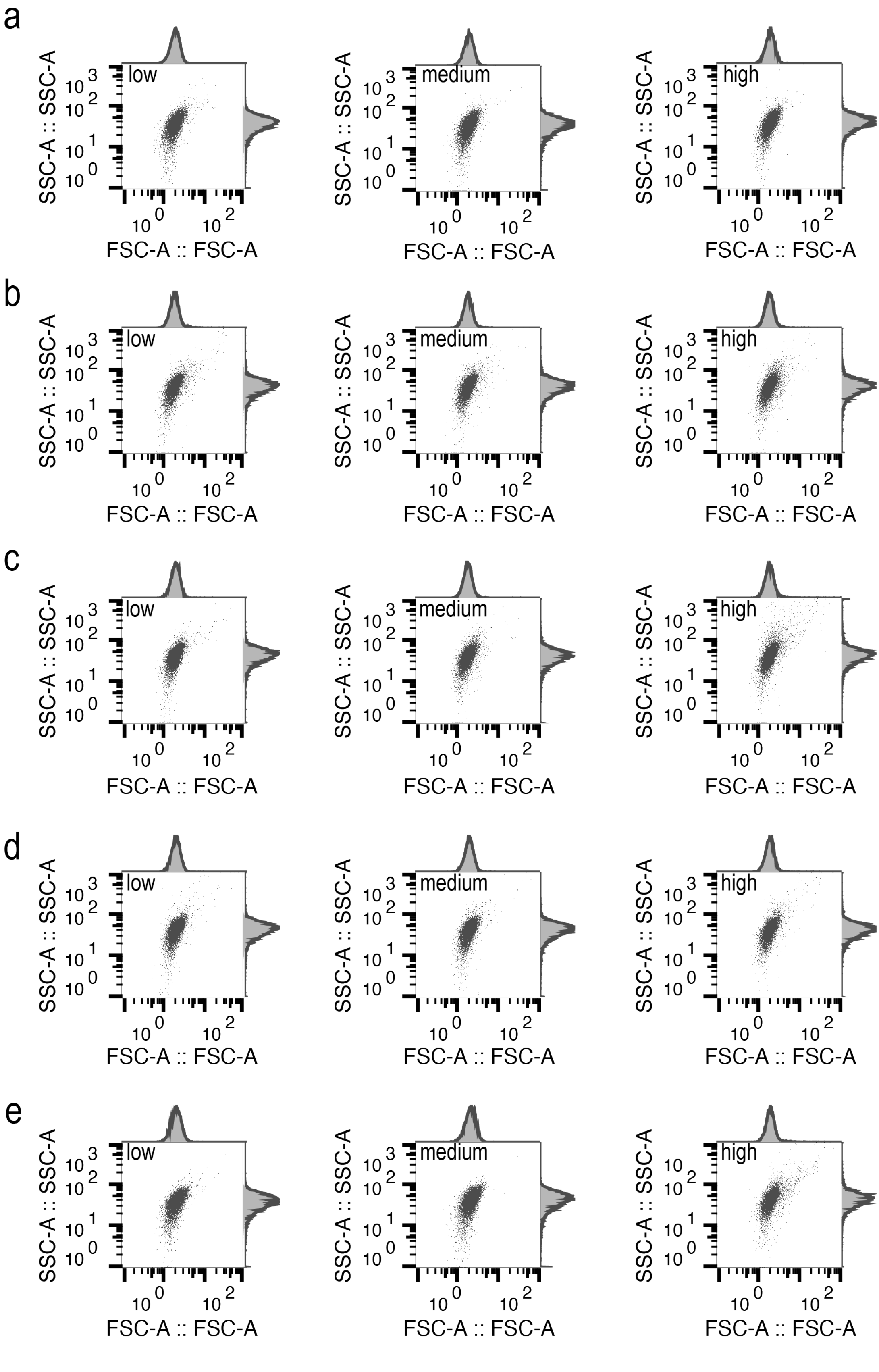

Supplementary Figure 14. Flow cytometry dot plots used to identify the *E. coli* population of SEGA strains with BBa_J23100 based on forward scattered (FSC) and side scattered (SSC) light. The experimentaly threshold was set to 3000. No further gating strategy was applied and therefore no cells excluded from the experiment. Data are shown for low (left), medium (middle) and high (right) expressing TIR variants harbouring the middle gadgets BCD **(a)**, Ubiquitin **(b)**, TEV **(c)**, PROTi **(d)**, PelBss **(e)** and the BBa_J23100 promoter. Raw data can be found on FlowRepository.org under the Repository ID FR-FCM-Z445.

Supplementary Figure 15.

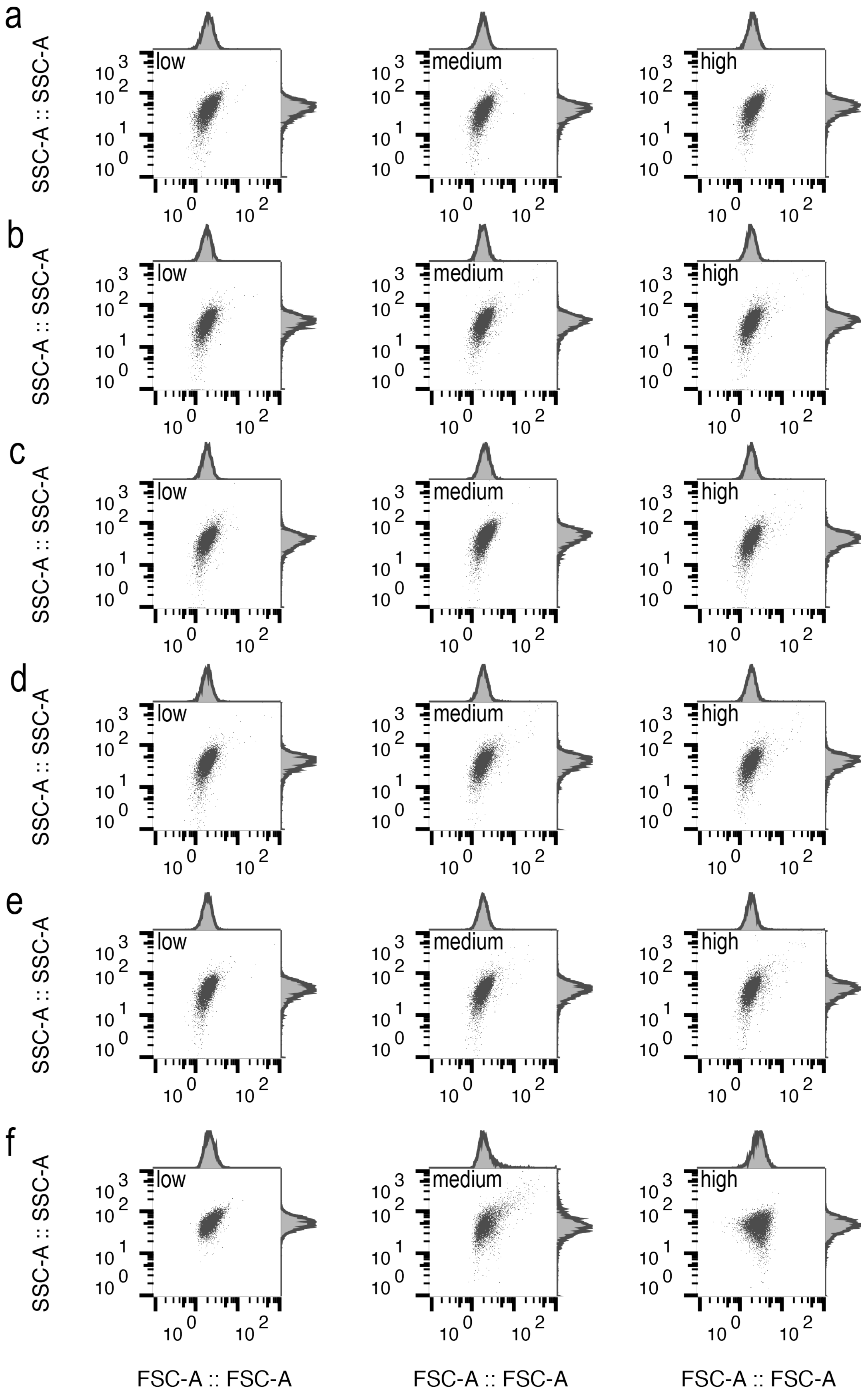

Supplementary Figure 15. Flow cytometry dot plots used to identify the *E. coli* population of SEGA strains with Ptrc based on forward scattered (FSC) and side scattered (SSC) light.
The experimentaly threshold was set to 3000. No further gating strategy was applied and therefore no cells excluded from the experiment. Data are shown for low (left), medium (middle) and high (right) expressing TIR variants harbouring the middle gadgets BCD **(a)**, Ubiquitin **(b)**, TEV **(c)**, PROTi **(d)**, PelBss **(e)**, YebF **(f)** and the *trc* promoter. Raw data can be found on FlowRepository.org under the Repository ID FR-FCM-Z445.

Supplementary Figure 16.

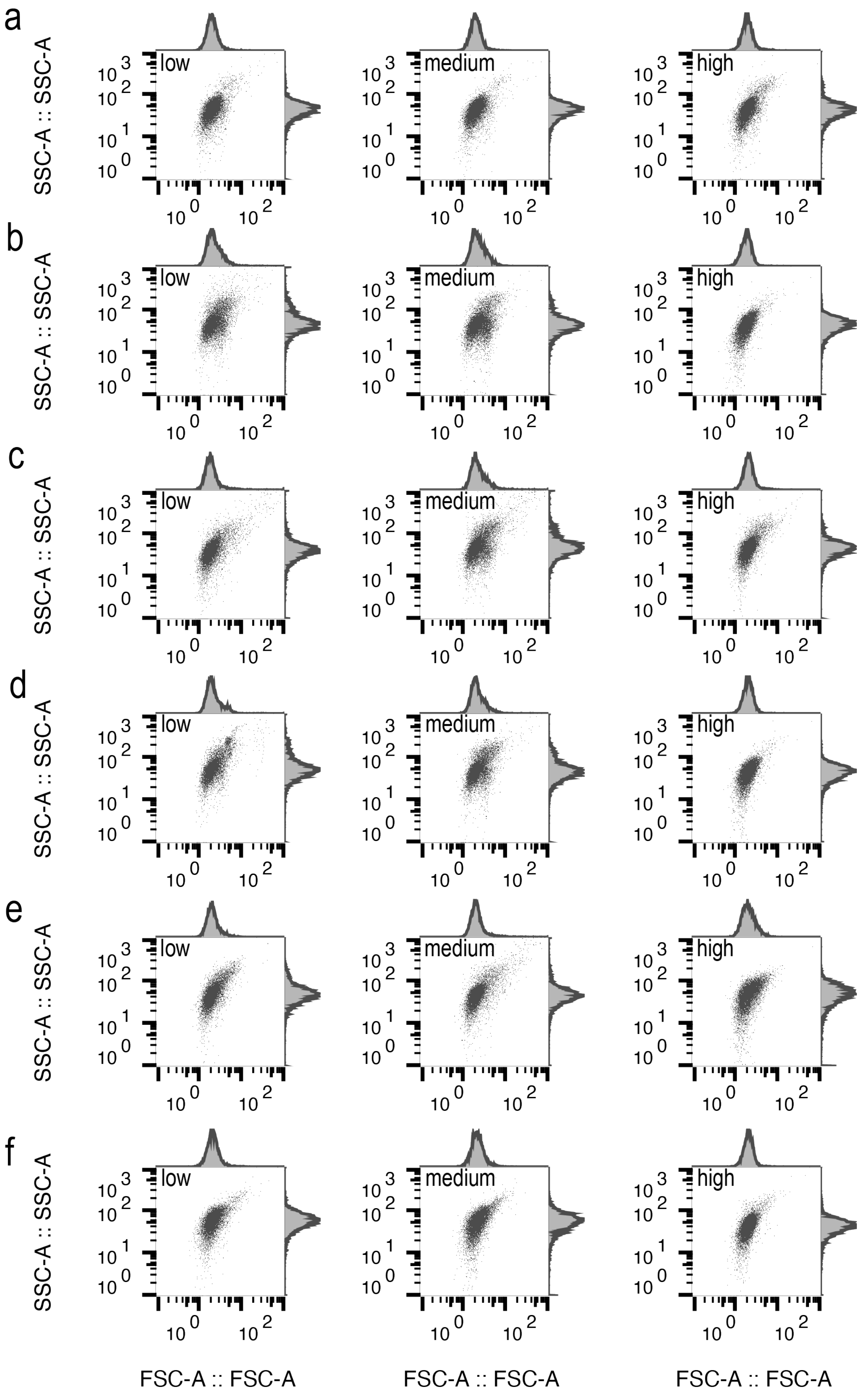

Supplementary Figure 16. Flow cytometry dot plots used to identify the *E. coli* population of SEGA strains with PT7 based on forward scattered (FSC) and side scattered (SSC) light. The experimentaly threshold was set to 3000. No further gating strategy was applied and therefore no cells excluded from the experiment. Data are shown for low (left), medium (middle) and high (right) expressing TIR variants harbouring the middle gadgets BCD **(a)**, Ubiquitin **(b)**, TEV **(c)**, PROTi **(d)**, PelBss **(e)**, YebF **(f)** and the T7 promoter. Raw data can be found on FlowRepository.org under the Repository ID FR-FCM-Z445.

Supplementary Figure 17.

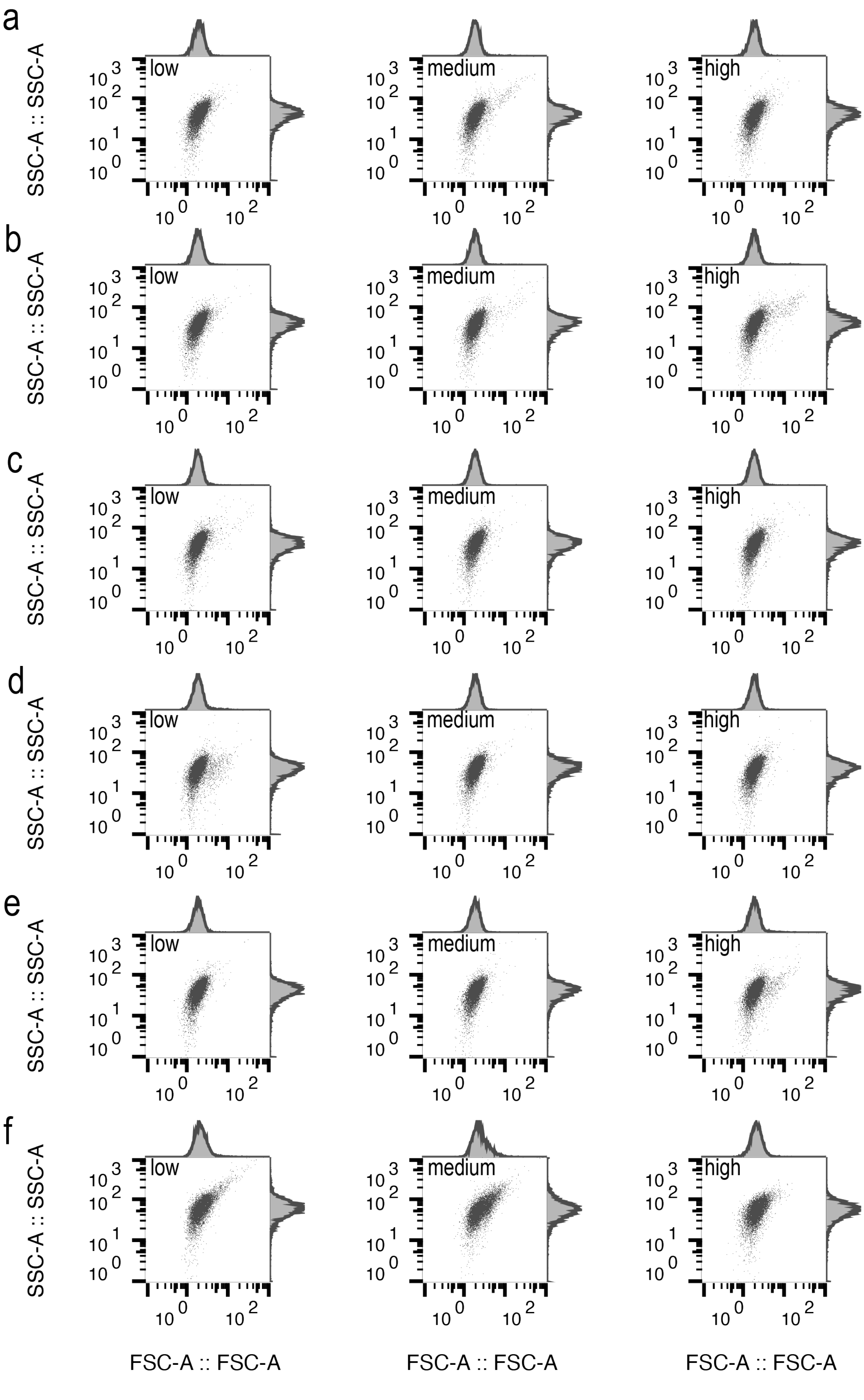

Supplementary Figure 17. Flow cytometry dot plots used to identify the *E. coli* population of SEGA strains with PrhaBAD based on forward scattered (FSC) and side scattered (SSC) light. The experimentaly threshold was set to 3000. No further gating strategy was applied and therefore no cells excluded from the experiment. Data are shown for low (left), medium (middle) and high (right) expressing TIR variants harbouring the middle gadgets BCD **(a)**, Ubiquitin **(b)**, TEV **(c)**, PROTi **(d)**, PelBss **(e)**, YebF **(f)** and the *rhaBAD* promoter. Raw data can be found on FlowRepository.org under the Repository ID FR-FCM-Z445.

Supplementary Figure 18.

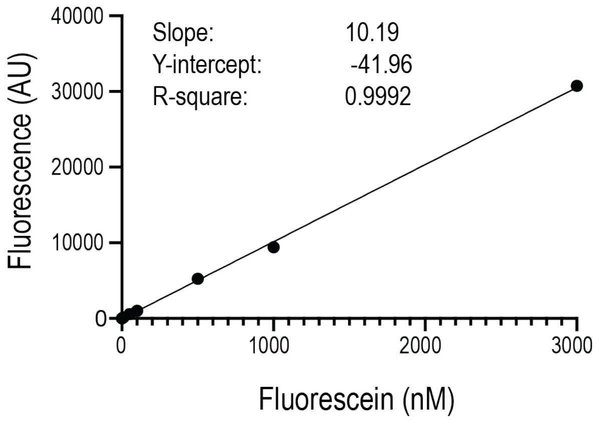

Supplementary Figure 18. Exemplary fluorescein standard curved used for GFP fluorescence normalization.

To normalize GFP fluorescence, a fluorescein standard curve was included in microtiter plate experiments. Fluorescein concentrations used were: 0 nM, 10 nM, 50 nM, 100 nM, 500 nM, 1000 nM and 3000 nM. A linear regression was made using GraphPad Prism. The presented data corresponds to the experiments shown in Figure 3e. Equivalent normalizations were performed for all microplate reader measurements of GFP fluorescence that are presented in the manuscript.

Supplementary Table 1.

Supplementary Table 1. Strains used in this study

Strain	Genotype	Source
**Unengineered strains**		
E. coli NEB5α	fhuA2 Δ(argF-lacZ)U169 *phoA glnV44* Φ80 Δ(*lacZ*)M15 *gyrA*96 *recA*1 *relA*1 *endA*1 *thi*-1 *hsdR*17	*a*
E. coli MG1655	K-12 F– λ– *ilvG– rfb*-50 *rph*-1	Lab stock
E. coli MG1655(DE3)	K-12 F– λ– *ilvG– rfb*-50 *rph*-1 λ(DE3)	[4]
E. coli JW0262-3	BW25113[b] *yagF::kanR*	[5]
E. coli JW3964-1	BW25113[b] *hupA::kanR*	[5]
E. coli JW2012-1	BW25113[b] *wbbL::kanR*	[5]
E. coli JW0302-1	BW25113[b] *ykgH::kanR*	[5]
E. coli JW1512-1	BW25113[b] *tam::kanR*	[5]
E. coli JW0334-1	BW25113[b] *lacY::kanR*	[5]
E. coli JW5611-1	BW25113[b] *atpI::kanR*	[5]
E. coli JW5126-1	BW25113[b] *ycbX::kanR*	[5]
E. coli JW2218-1	BW25113[b] *atoB::kanR*	[5]

Strain	Genotype	Source
E. coli MG1655 "Marionette-Wild"	K-12 F– λ– ilvG– rfb-50 rph-1 glvC::Marionette-cluster	[2]
**Base strains for SEGA landing pad assembly**		
SIJ19	*E. coli* K12 MG1655, *glmS-pstS* :: J23100-*gfp*	[6]
MG1655 – $TetA^{OPT}$	*E. coli* K12, *glmS-pstS* :: J23100-BCD-*gfp-tetA*	Nørholm lab, unpublished
**Strains from the SEGA strain collection.** For details of the SEGA modules and additional strains, see Supplementary File 2		
SEGA001	*E. coli* K12, *glmS-pstS* :: J23100-3'*tetA*	this study
SEGA002	*E. coli* K12, *glmS-pstS* :: Ptrc-3'*tetA*	this study
SEGA003	*E. coli* K12, *glmS-pstS* :: PRhaBAD-3'*tetA*	this study
SEGA004	*E. coli* K12, *glmS-pstS* :: PT7-3'*tetA*	this study
SEGA005	*E. coli* K12, *glmS-pstS* :: J23100-low-BCD-*gfp-tetA*	this study
SEGA006	*E. coli* K12, *glmS-pstS* :: J23100-medium-BCD-*gfp-tetA*	this study
SEGA007	*E. coli* K12, *glmS-pstS* :: J23100-high-BCD-*gfp-tetA*	this study
SEGA008	*E. coli* K12, *glmS-pstS* :: J23100-low-Ubiquitin-*gfp-tetA*	this study
SEGA009	*E. coli* K12, *glmS-pstS* :: J23100-medium-Ubiquitin-*gfp-tetA*	this study
SEGA010	*E. coli* K12, *glmS-pstS* :: J23100-high-Ubiquitin-*gfp-tetA*	this study
SEGA011	*E. coli* K12, *glmS-pstS* :: J23100-low-TEV-*gfp-tetA*	this study
SEGA012	*E. coli* K12, *glmS-pstS* :: J23100-medium-TEV-*gfp-tetA*	this study
SEGA013	*E. coli* K12, *glmS-pstS* :: J23100-high-TEV-*gfp-tetA*	this study
SEGA014	*E. coli* K12, *glmS-pstS* :: J23100-low-PROTi-*gfp-tetA*	this study
SEGA015	*E. coli* K12, *glmS-pstS* :: J23100-medium-PROTi-*gfp-tetA*	this study
SEGA016	*E. coli* K12, *glmS-pstS* :: J23100-high-PROTi-*gfp-tetA*	this study
SEGA017	*E. coli* K12, *glmS-pstS* :: J23100-low-PelB-*sfgfp-tetA*	this study
SEGA018	*E. coli* K12, *glmS-pstS* :: J23100-medium-PelB-*sfgfp-tetA*	this study
SEGA019	*E. coli* K12, *glmS-pstS* :: J23100-high-PelB-*sfgfp-tetA*	this study
SEGA020	*E. coli* K12, *glmS-pstS* :: Ptrc-low-BCD-*gfp-tetA*	this study
SEGA021	*E. coli* K12, *glmS-pstS* :: Ptrc-medium-BCD-*gfp-tetA*	this study
SEGA022	*E. coli* K12, *glmS-pstS* :: Ptrc-high-BCD-*gfp-tetA*	this study
SEGA023	*E. coli* K12, *glmS-pstS* :: Ptrc-low-Ubiquitin-*gfp-tetA*	this study
SEGA024	*E. coli* K12, *glmS-pstS* :: Ptrc-medium-Ubiquitin-*gfp-tetA*	this study
SEGA025	*E. coli* K12, *glmS-pstS* :: Ptrc-high-Ubiquitin-*gfp-tetA*	this study
SEGA026	*E. coli* K12, *glmS-pstS* :: Ptrc-low-TEV-*gfp-tetA*	this study
SEGA027	*E. coli* K12, *glmS-pstS* :: Ptrc-medium-TEV-*gfp-tetA*	this study
SEGA028	*E. coli* K12, *glmS-pstS* :: Ptrc-high-TEV-*gfp-tetA*	this study
SEGA029	*E. coli* K12, *glmS-pstS* :: Ptrc-low-PROTi-*gfp-tetA*	this study
SEGA030	*E. coli* K12, *glmS-pstS* :: Ptrc-medium-PROTi-*gfp-tetA*	this study
SEGA031	*E. coli* K12, *glmS-pstS* :: Ptrc-high-PROTi-*gfp-tetA*	this study
SEGA032	*E. coli* K12, *glmS-pstS* :: Ptrc-low-PelB-*sfgfp-tetA*	this study
SEGA033	*E. coli* K12, *glmS-pstS* :: Ptrc-medium-PelB-*sfgfp-tetA*	this study

**Strain**	**Genotype**	**Source**
SEGA034	*E. coli* K12, *glmS-pstS* :: Ptrc-high-PelB-*sfgfp-tetA*	this study
SEGA035	*E. coli* K12, *glmS-pstS* :: Ptrc-low-YebF-*sfgfp-tetA*	this study
SEGA036	*E. coli* K12, *glmS-pstS* :: Ptrc-medium-YebF-*sfgfp-tetA*	this study
SEGA037	*E. coli* K12, *glmS-pstS* :: Ptrc-high-YebF-*sfgfp-tetA*	this study
SEGA038	*E. coli* K12, *glmS-pstS* :: PrhaBAD-low-BCD-*gfp-tetA*	this study
SEGA039	*E. coli* K12, *glmS-pstS* :: PrhaBAD-medium-BCD-*gfp-tetA*	this study
SEGA040	*E. coli* K12, *glmS-pstS* :: PrhaBAD-high-BCD-*gfp-tetA*	this study
SEGA041	*E. coli* K12, *glmS-pstS* :: PrhaBAD-low-Ubiquitin-*gfp-tetA*	this study
SEGA042	*E. coli* K12, *glmS-pstS* :: PrhaBAD-medium-Ubiquitin-*gfp-tetA*	this study
SEGA043	*E. coli* K12, *glmS-pstS* :: PrhaBAD-high-Ubiquitin-*gfp-tetA*	this study
SEGA044	*E. coli* K12, *glmS-pstS* :: PrhaBAD-low-TEV-*gfp-tetA*	this study
SEGA045	*E. coli* K12, *glmS-pstS* :: PrhaBAD-medium-TEV-*gfp-tetA*	this study
SEGA046	*E. coli* K12, *glmS-pstS* :: PrhaBAD-high-TEV-*gfp-tetA*	this study
SEGA047	*E. coli* K12, *glmS-pstS* :: PrhaBAD-low-PROTi-*gfp-tetA*	this study
SEGA048	*E. coli* K12, *glmS-pstS* :: PrhaBAD-medium-PROTi-*gfp-tetA*	this study
SEGA049	*E. coli* K12, *glmS-pstS* :: PrhaBAD-high-PROTi-*gfp-tetA*	this study
SEGA050	*E. coli* K12, *glmS-pstS* :: PrhaBAD-low-PelB-*sfgfp-tetA*	this study
SEGA051	*E. coli* K12, *glmS-pstS* :: PrhaBAD-medium-PelB-*sfgfp-tetA*	this study
SEGA052	*E. coli* K12, *glmS-pstS* :: PrhaBAD-high-PelB-*sfgfp-tetA*	this study
SEGA053	*E. coli* K12, *glmS-pstS* :: PrhaBAD-low-YebF-*sfgfp-tetA*	this study
SEGA054	*E. coli* K12, *glmS-pstS* :: PrhaBAD-medium-YebF-*sfgfp-tetA*	this study
SEGA055	*E. coli* K12, *glmS-pstS* :: PrhaBAD-high-YebF-*sfgfp-tetA*	this study
SEGA056	*E. coli* K12 (DE3), *glmS-pstS* :: PT7-low-BCD-*gfp-tetA*	this study
SEGA057	*E. coli* K12 (DE3), *glmS-pstS* :: PT7-medium-BCD-*gfp-tetA*	this study
SEGA058	*E. coli* K12 (DE3), *glmS-pstS* :: PT7-high-BCD-*gfp-tetA*	this study
SEGA059	*E. coli* K12 (DE3), *glmS-pstS* :: PT7-low-Ubiquitin-*gfp-tetA*	this study
SEGA060	*E. coli* K12 (DE3), *glmS-pstS* :: PT7-medium-Ubiquitin-*gfp-tetA*	this study
SEGA061	*E. coli* K12 (DE3), *glmS-pstS* :: PT7-high-Ubiquitin-*gfp-tetA*	this study
SEGA062	*E. coli* K12 (DE3), *glmS-pstS* :: PT7-low-TEV-*gfp-tetA*	this study
SEGA063	*E. coli* K12 (DE3), *glmS-pstS* :: PT7-medium-TEV-*gfp-tetA*	this study
SEGA064	*E. coli* K12 (DE3), *glmS-pstS* :: PT7-high-TEV-*gfp-tetA*	this study
SEGA065	*E. coli* K12 (DE3), *glmS-pstS* :: PT7-low-PROTi-*gfp-tetA*	this study
SEGA066	*E. coli* K12 (DE3), *glmS-pstS* :: PT7-medium-PROTi-*gfp-tetA*	this study
SEGA067	*E. coli* K12 (DE3), *glmS-pstS* :: PT7-high-PROTi-*gfp-tetA*	this study
SEGA068	*E. coli* K12 (DE3), *glmS-pstS* :: PT7-low-PelB-*sfgfp-tetA*	this study
SEGA069	*E. coli* K12 (DE3), *glmS-pstS* :: PT7-medium-PelB-*sfgfp-tetA*	this study
SEGA070	*E. coli* K12 (DE3), *glmS-pstS* :: PT7-high-PelB-*sfgfp-tetA*	this study
SEGA071	*E. coli* K12 (DE3), *glmS-pstS* :: PT7-low-YebF-*sfgfp-tetA*	this study
SEGA072	*E. coli* K12 (DE3), *glmS-pstS* :: PT7-medium-YebF-*sfgfp-tetA*	this study
SEGA073	*E. coli* K12 (DE3), *glmS-pstS* :: PT7-high-YebF-*sfgfp-tetA*	this study

Strain	Genotype	Source
SEGA075	*E. coli* K12, *glmS-pstS* :: PSal-high-BCD-*gfp-tetA*	this study
SEGA077	*E. coli* K12, *glmS-pstS* :: PTet-high-BCD-*gfp-tetA*	this study
SEGA080	*E. coli* K12 *ΔgalK, glmS-pstS ::* PrhaBAD-high-BCD-*gfp-tetA*	this study
SEGA096	*E. coli* K12, *glmS-pstS* :: J23100-low-BCD-3'*tetA*	this study
SEGA097	*E. coli* K12, *glmS-pstS* :: J23100-high-BCD-3'*tetA*	this study
SEGA098	*E. coli* K12, *glmS-pstS* :: J23100-high-BCD-*gfp*-3'*tetA*	this study
SEGA099	*E. coli* K12, *glmS-pstS* :: PTrc-low-BCD-3'*tetA*	this study
SEGA100	*E. coli* K12, *glmS-pstS* :: PTrc-high-BCD-3'*tetA*	this study
SEGA101	*E. coli* K12, *glmS-pstS* :: PRhaBAD-low-BCD-3'*tetA*	this study
SEGA102	*E. coli* K12, *glmS-pstS* :: PRhaBAD-high-BCD-3'*tetA*	this study
SEGA103	*E. coli* K12 (DE3), *glmS-pstS* :: PT7-low-BCD-3'*tetA*	this study
SEGA104	*E. coli* K12 (DE3), *glmS-pstS* :: PT7-high-BCD-3'*tetA*	this study
**SEGA strains with cargo integrations**		
SEGA005-RFP	*E. coli* K12, *glmS-pstS* :: J23100-low-BCD-*rfp*	this study
SEGA006-RFP	*E. coli* K12, *glmS-pstS* :: J23100-medium-BCD-*rfp*	this study
SEGA007-RFP	*E. coli* K12, *glmS-pstS* :: J23100-high-BCD-*rfp*	this study
SEGA008-RFP	*E. coli* K12, *glmS-pstS* :: J23100-low-Ubiquitin-*rfp*	this study
SEGA009-RFP	*E. coli* K12, *glmS-pstS* :: J23100-medium-Ubiqutin-*rfp*	this study
SEGA010-RFP	*E. coli* K12, *glmS-pstS* :: J23100-high-Ubiquitin-*rfp*	this study
SEGA011-RFP	*E. coli* K12, *glmS-pstS* :: J23100-low-TEV-*rfp*	this study
SEGA012-RFP	*E. coli* K12, *glmS-pstS* :: J23100-medium-TEV-*rfp*	this study
SEGA013-RFP	*E. coli* K12, *glmS-pstS* :: J23100-high-TEV-*rfp*	this study
SEGA014-RFP	*E. coli* K12, *glmS-pstS* :: J23100-low-PROTi-*rfp*	this study
SEGA015-RFP	*E. coli* K12, *glmS-pstS* :: J23100-medium-PROTi-*rfp*	this study
SEGA016-RFP	*E. coli* K12, *glmS-pstS* :: J23100-high-PROTi-*rfp*	this study
SEGA017-RFP	*E. coli* K12, *glmS-pstS* :: J23100-low-PelB-*rfp*	this study
SEGA018-RFP	*E. coli* K12, *glmS-pstS* :: J23100-medium-PelB-*rfp*	this study
SEGA019-RFP	*E. coli* K12, *glmS-pstS* :: J23100-high-PelB-*rfp*	this study
SEGA007-CrtE-truncTetA	*E. coli* K12, *glmS-pstS* :: J23100 - high - BCD - *crtE* - 3'*tetA*	this study
SEGA007-CrtEB-TetA	*E. coli* K12, *glmS-pstS* :: J23100 - high - BCD - *crtEB* - *tetA*	this study
SEGA007-CrtEBI-truncTetA	*E. coli* K12, *glmS-pstS* :: J23100 - high - BCD - *crtEBI* - 3'*tetA*	this study
SEGA007- CrtEBIY-TetA	*E. coli* K12, *glmS-pstS* :: J23100 - high - BCD - *crtEBIY* - *tetA*	this study
SEGA007-CrtEBIY-TetA (*)	*E. coli* K12, *glmS-pstS* :: J23100 - high - BCD - *crtEBIY* - *tetA* (constructed by one step integration, same genotype as above)	this study
SEGA020-YidC	*E. coli* K12, *glmS-pstS* :: Ptrc-low-BCD-*yidC-gfp-tetA*	this study
SEGA022-YidC	*E. coli* K12, *glmS-pstS* :: Ptrc-high-BCD-*yidC-gfp-tetA*	this study
SEGA038-YidC	*E. coli* K12, *glmS-pstS* :: PrhaBAD-low-BCD-*yidC-gfp-tetA*	this study
SEGA040-YidC	*E. coli* K12, *glmS-pstS* :: PrhaBAD-high-BCD-*yidC-gfp-tetA*	this study

Strain	Genotype	Source
SEGA056-YidC	*E. coli* K12 (DE3), *glmS-pstS* :: PT7-low-BCD-*yidC-gfp-tetA*	this study
SEGA058-YidC	*E. coli* K12 (DE3), *glmS-pstS* :: PT7-high-BCD-*yidC-gfp-tetA*	this study
SEGA020-Nanobody	*E. coli* K12, *glmS-pstS* :: Ptrc-low-BCD-*Nanobody*	this study
SEGA021-Nanobody	*E. coli* K12, *glmS-pstS* :: Ptrc-medium-BCD-*Nanobody*	this study
SEGA022-Nanobody	*E. coli* K12, *glmS-pstS* :: Ptrc-high-BCD-*Nanobody*	this study
**Marionette background strain / alternative promoter architecture**		
E. coli "Marionette-Wild" - PSal	Marionette-Wild, *glmS-pstS* :: PSalTTC-RiboJ-high-BCD-*gfp-tetA*	this study
E. coli "Marionette-Wild" - PTet	Marionette-Wild, *glmS-pstS* :: PTet-RiboJ-high-BCD-*gfp-tetA*	this study
SEGA - PSal - truncTetA	*E. coli* K12, *glmS-pstS* :: PSal-3'*tetA*	this study
SEGA - PTet - truncTetA	*E. coli* K12, *glmS-pstS* :: PTet-3'*tetA*	this study
**SEGA landing pad integrated in different genome loci**		
SEGA - *yagF* - J23100-BCD-low	*E. coli* K12, *yagF(-yagG)* :: J23100-low-BCD-*gfp-tetA*	this study
SEGA - *yagF* - J23100-BCD-high	*E. coli* K12, *yagF(-yagG)* :: J23100-high-BCD-*gfp-tetA*	this study
SEGA - *hupA* - J23100-BCD-low	*E. coli* K12, *(yjaG-)hupA* :: J23100-low-BCD-*gfp-tetA*	this study
SEGA - *hupA* - J23100-BCD-high	*E. coli* K12, *(yjaG-)hupA* :: J23100-high-BCD-*gfp-tetA*	this study
SEGA - *wbbL* - J23100-BCD-low	*E. coli* K12, *wbbL(-gnd)* :: J23100-low-BCD-*gfp-tetA*	this study
SEGA - *wbbL* - J23100-BCD-high	*E. coli* K12, *wbbL(-gnd)* :: J23100-high-BCD-*gfp-tetA*	this study
SEGA - *ykgH* - J23100-BCD-low	*E. coli* K12, *ykgH(-ykgG) ::* J23100-low-BCD-*gfp-tetA*	this study
SEGA - *ykgH* - J23100-BCD-high	*E. coli* K12, *ykgH(-ykgG) ::* J23100-high-BCD-*gfp-tetA*	this study
SEGA - *tam* - J23100-BCD-low	*E. coli* K12, *tam(-yneE) ::* J23100-low-BCD-*gfp-tetA*	this study
SEGA - *tam* - J23100-BCD-high	*E. coli* K12, *tam(-yneE) ::* J23100-high-BCD-*gfp-tetA*	this study
SEGA - *lacZ* - J23100-BCD-low	*E. coli* K12, *(lacI-)lacZ ::* J23100-low-BCD-*gfp-tetA*	this study
SEGA - *lacZ* - J23100-BCD-high	*E. coli* K12, *(lacI-)lacZ ::* J23100-high-BCD-*gfp-tetA*	this study
SEGA - *rsmG-atpI* - J23100-BCD-low	*E. coli* K12, *rsmG-atpI* :: J23100-low-BCD-*gfp-tetA*	this study
SEGA - *rsmG-atpI* - J23100-BCD-high	*E. coli* K12, *rsmG-atpI* :: J23100-high-BCD-*gfp-tetA*	this study
SEGA - *recA* - J23100-BCD-low	*E. coli* K12, *(pncC-)recA ::* J23100-low-BCD-*gfp-tetA*	this study
SEGA - *recA* - J23100-BCD-high	*E. coli* K12, *(pncC-)recA ::* J23100-high-BCD-*gfp-tetA*	this study
SEGA - *ycbX* - J23100-BCD-low	*E. coli* K12, *ycbX(-zapC)* :: J23100-low-BCD-*gfp-tetA*	this study

Strain	Genotype	Source
SEGA - *ycbX* - J23100-BCD-high	*E. coli* K12, *ycbX(-zapC)* :: J23100-high-BCD-*gfp-tetA*	this study
SEGA - *atoB* - J23100-BCD-low	*E. coli* K12, *atoB(-yfaP)* :: J23100-low-BCD-*gfp-tetA*	this study
SEGA - *atoB* - J23100-BCD-high	*E. coli* K12, *atoB(-yfaP)* :: J23100-high-BCD-*gfp-tetA*	this study
KEIO *ΔyagF* :: J23100-BCD-low	*E. coli* JW0262-3 (*ΔyagF*), *kanR* :: J23100-low-BCD-*gfp-tetA*	this study
KEIO *ΔyagF* :: J23100-BCD-high	*E. coli* JW0262-3 (*ΔyagF*), *kanR* :: J23100-high-BCD-*gfp-tetA*	this study
KEIO *ΔhupA* :: J23100-BCD-low	*E. coli* JW3964-1 (*ΔhupA*), *kanR* :: J23100-low-BCD-*gfp-tetA*	this study
KEIO *ΔhupA* :: J23100-BCD-high	*E. coli* JW3964-1 (*ΔhupA*), *kanR* :: J23100-high-BCD-*gfp-tetA*	this study
KEIO *ΔwbbL ::* J23100-BCD-low	*E. coli* JW2012-1 (*ΔwbbL*), *kanR* :: J23100-low-BCD-*gfp-tetA*	this study
KEIO *ΔwbbL* :: J23100-BCD-high	*E. coli* JW2012-1 (*ΔwbbL*), *kanR* :: J23100-high-BCD-*gfp-tetA*	this study
KEIO *ΔykgH* :: J23100-BCD-low	*E. coli* JW0302-1 (*ΔykgH*), *kanR* :: J23100-low-BCD-*gfp-tetA*	this study
KEIO *ΔykgH* :: J23100-BCD-high	*E. coli* JW0302-1 (*ΔykgH*), *kanR* :: J23100-high-BCD-*gfp-tetA*	this study
KEIO *Δtam* :: J23100-BCD-low	*E. coli* JW1512-1 (*Δtam*), *kanR* :: J23100-low-BCD-*gfp-tetA*	this study
KEIO *Δtam* :: J23100-BCD-high	*E. coli* JW1512-1 (*Δtam*), *kanR* :: J23100-high-BCD-*gfp-tetA*	this study
KEIO *ΔlacY* :: J23100-BCD-low	*E. coli* JW0334-1 (*ΔlacY*), *kanR* :: J23100-low-BCD-*gfp-tetA*	this study
KEIO ΔlacY :: J23100-BCD-high	*E. coli* JW0334-1 (*ΔlacY*), *kanR* :: J23100-high-BCD-*gfp-tetA*	this study
KEIO *ΔatpI* :: J23100-BCD-low	*E. coli* JW5611-1 (*ΔatpI*), *kanR* :: J23100-low-BCD-*gfp-tetA*	this study
KEIO *ΔatpI* :: J23100-BCD-high	*E. coli* JW5611-1 (*ΔatpI*), *kanR* :: J23100-high-BCD-*gfp-tetA*	this study
KEIO *ΔycbX* :: J23100-BCD-low	*E. coli* JW5126-1 (*ΔycbX*), *kanR* :: J23100-low-BCD-*gfp-tetA*	this study
KEIO *ΔycbX* :: J23100-BCD-high	*E. coli* JW5126-1 (*ΔycbX*), *kanR* :: J23100-high-BCD-*gfp-tetA*	this study
KEIO *ΔatoB* :: J23100-BCD-low	*E. coli* JW2218-1 (*ΔatoB*), *kanR* :: J23100-low-BCD-*gfp-tetA*	this study
KEIO *ΔatoB* :: J23100-BCD-high	*E. coli* JW2218-1 (*ΔatoB*), *kanR* :: J23100-high-BCD-*gfp-tetA*	this study

[a]NEB, Ipswich, MA, USA; [b] BW25113: *Δ*(*araB–D*)567 *Δ(rhaD–B)*568 *ΔlacZ*4787(::rrnB-3) *hsdR514 rph-1*

Supplementary Table 2.

Supplementary Table 2. Plasmids used in this study

Plasmid	Property	Source/Reference
pSIM19	vector encoding *gam, beta, exo* for λ-Red recombination, SpecR, pSC101 ori (temperature sensitive)	[7]
pET28a-AraH-GFP	vector encoding *araH-gfp* under control of PT7, KanR, pBR322 ori	[8]
pPROTi	vector encoding tev protease under control of PRhaBAD, CmR, pBBR1 ori	[9]
pET-Duet1-Ptrc-GFPopt	vector encoding *gfp* under control of Ptrc, AmpR, pBR322 ori	[10]
pET39b-Ub(His10)	vector encoding ubiquitin with an internal His10-tag, KanR, pBR322 ori	provided by Lars Ellgaard
pmskl2	vector encoding *mCherry* and *gfp*, KanR, pSC101 ori	provided by Michael Schantz Klausen
pZE21-RFP	vector encoding *rfp*, KanR, pBR322 ori	provided by Andreas Porse
pET28a-YidC-GFP	vector encoding *yidC-gfp*, KanR, pBR322 ori	[11]
pSEVA36-sl3-crtEBIY	vector encoding *P. ananatis crtEBIY*, CmR, p15A ori	[12]
pET28a-Nanobody-hp6-AmpR	vector encoding a Nanobody targeted towards GFP, KanR, pBR322 ori	[1]
pET28a-*sfgfp*	vector encoding *sfgfp*, KanR, pBR322 ori	provided by Cristina Hernández-Rollán
pSEVA27-sl3-ccdB	vector encoding *ccdB*, KanR, pSC101 ori	[12]
pSEVA27-sl3-J23100-BCD-*gfp*	vector encoding BCD-*gfp-5'tetA*, KanR, pSC101 ori	this study
pSEVA27-sl3-J23100-Ub(His10)-*gfp*	vector encoding Ub(His10)-*gfp-5'tetA*, KanR, pSC101 ori	this study
pSEVA27-sl3-J23100-TEV-*gfp*	vector encoding TEVsite-*gfp-5'tetA*, KanR, pSC101 ori	this study
pSEVA27-sl3-J23100-PROTi-*gfp*	vector encoding PROTi-tag-*gfp-5'tetA*, KanR, pSC101 ori	this study
pSEVA27-sl3-J23100-PelBss-*sfgfp*	vector encoding PelBss-*sfgfp-5'tetA*, KanR, pSC101 ori	this study
pSEVA27-sl3-J23100-YebFss-*sfgfp*	vector encoding YebFss-*sfgfp-5'tetA*, KanR, pSC101 ori	this study
pSEVA27-sl3-PrhaBAD-YebF(TIR low)-*sfgfp*	vector encoding *yebF-sfgfp-5'tetA* with TIR sequence for low expression levels, KanR, pSC101 ori	this study
pSEVA27-sl3-PrhaBAD-YebF(TIR medium)-*sfgfp*	vector encoding *yebF-sfgfp-5'tetA* with TIR sequence for medium expression levels, KanR, pSC101 ori	this study

Plasmid	Property	Source/Reference
pSEVA27-sl3-PrhaBAD-YebF(TIR high)-*sfgfp*	vector encoding *yebF-sfgfp-5'tetA* with TIR sequence for high expression levels, KanR, pSC101 ori	this study
pSEVA27-sl3-PT7-*yidC-gfp-5'tetA*	vector encoding *yidC-gfp*, KanR, pSC101 ori	this study
pSEVA27-sl3-*crtB-5'tetA*	vector encoding *crtB-5'tetA*, KanR, pSC101 ori	this study
pSEVA27-sl3-*crtIY-5'tetA*	vector encoding *crtIY-5'tetA*, KanR, pSC101 ori	this study
pSEVA27-sl3-*crtEBIY-5'tetA*	vector encoding *crtEBIY-5'tetA,* KanR, pSC101 ori	this study
pAJM.771	vector encoding PSalTTC-RiboJ-*yfp-nahR*, KanR, p15A ori	[2]
pAJM.011	vector encoding PTet-RiboJ-*yfp-tetR*, KanR, p15A ori	[2]
pAJM.771-NahR-PSal	vector encoding *nahR*-PSalTTC-RiboJ-*yfp*, KanR, p15A ori – modified from pAJM.771 to change positioning of the *nahR* regulator gene	this study
pAJM.011-TetR-Ptet	vector encoding *tetR*-Ptet-RiboJ-*yfp*, KanR, p15A ori – modified from pAJM.011 to change positioning of the *tetR* regulator gene	this study

Supplementary Table 3.

Supplementary Table 3. Oligonucleotides used in this study

Number	Sequence	Description
Oligonucleotides for genome integration		
101	CATCTAATTCAACAAGAATTGGCACAACTCCAGTGAAAAGTTCTTCTCCTTTGCTCATTAGAAAACCTCCTTAGCATGATTAAGATGTTTCAGTACGAAAATNGCYTTCATNNNNNNTTTCTCCTCTTTAATCTCTAGTAGCTAGCACTGTACCTAGGACTGAGC	Introducing BCD in front of *gfp* with CRISPR/Cas9
102	TTGTAACTGCTGCTGGGATTACACATGGCATGGATGAGCTCTACAAATGACCAATTATTGAAGGCCGCTAACGCGGCCTTTTTTTGTTTCTGGTCTCCCAAAAATTTATTTGCTTATTA	Original introduction of *tetA* on the genome
103	CTGATTTAAATAAGCGTTGATATTCAGTCAATTACAAACATTAATAACGAAGAGATGACAGAAAAATTTTAGAAATCATCCTTAGCGAAAGCTAAGGATTTTTTTTATCTGGACAATTGTCTCAGGTCGAGGTGGCCCGGCTC	Original introduction of *tetA* on the genome
105	ACCAGCCGCGTAACCTGGCAAAATCGGTTACGGTTGAGTAATAAATGGATCTCGGTACCAAATTCCAGAAAAGAGACGCTTTCGAGCGTCTTTTTTCGTTTTGGTCCGCGTTGCGCTTGACGGCTAG	Integration of upstream terminator L3S2P11
106	GATGCTGTCGGAATGGACGATATCCCGCAAGAGGCCCGGCAGTACTTTCTCCTCTTTAATCTCTAGTAGCTAGCACTGTACCTAGGACTG	tetA truncation on genome (J23100)
107	GATGCTGTCGGAATGGACGATATCCCGCAAGAGGCCCGGCAGTACTTTCTCCTCTTTAATCTCTAGTATACGACCAGTCTAAAAAGCGCC	PrhaBAD integration / *tetA* truncation
108	GATGCTGTCGGAATGGACGATATCCCGCAAGAGGCCCGGCAGTACTTTCTCCTCTTTAATCTCTAGTAGGAATTGTTATCCGCTCACAAT	Ptrc/PT7 integration / *tetA* truncation
109	CGTCTTTTTTCGTTTTGGTCCGCGTTGCGCTTAATCTTTCTGCGAATTGA	PrhaBAD integration / *tetA* truncation
110	AGAAAAGAGACGCTTTCGAGCGTCTTTTTTCGTTTTGGTCCGCGTTGCGCTCACTGCCCGCTTTCCAGTC	Ptrc/PT7 integration / *tetA* truncation
111	CGCTAGCAGCACGCCATAG	TIR transfer/ reestablish *tetA* (reverse)
112	ATCCAGGCGCTTTTTAGACTGGTCGTATACTAGAGATTAAAGAGGAGAAA	TIR transfer (specific to C1: PRhaBAD)
113	GAATTGTGAGCGGATAACAATTCCTACTAGAGATTAAAGAGGAG	TIR transfer (specific to C1: PT7 and PTrc)
114	TACTAGAGATTAAAGAGGAGAAA	Universal oligo for TIR transfer / Integration full YebF protein
115	CAGTCCTAGGTACAGTGCTAGCTACTAGAGATTAAAGAGGAGAAACGGGGGATGGAGAACCTGTACTTCCAGTTTCGTAG	Transfer of TEV TIR (low) to PROTi
116	CAGTCCTAGGTACAGTGCTAGCTACTAGAGATTAAAGAGGAGAAAAACTACATGGAAAACCTGTACTTCCAGTTTCGTAG	Transfer of TEV TIR (medium) to PROTi

Number	Sequence	Description
117	CAGTCCTAGGTACAGTGCTAGCTACTAGAGATTAAAGAGGAGAAAATATCGATGGAGAACCTGTACTTCCAGTTTCGTAG	Transfer of TEV TIR (high) to PROTi
118	CAGTCCTAGGTACAGTGCTAGCTACTAGAGATTAAAGAGGAGAAANNNNNNATGAARGCNATTTTCGTACTGAAACATCT	TIR library BCD
119	CAGTCCTAGGTACAGTGCTAGCTACTAGAGATTAAAGAGGAGAAANNNNNNATGCARATHTTCGTGAAAACACTGACCGG	TIR library Ubiquitin
120	CAGTCCTAGGTACAGTGCTAGCTACTAGAGATTAAAGAGGAGAAANNNNNNATGGARAAYCTGTACTTCCAGAGCAAAGG	TIR library TEV
121	CAGTCCTAGGTACAGTGCTAGCTACTAGAGATTAAAGAGGAGAAANNNNNNATGGARAAYCTGTACTTCCAGTTTCGTAG	TIR library PROTi
122	CAGTCCTAGGTACAGTGCTAGCTACTAGAGATTAAAGAGGAGAAANNNNNNATGAARTAYCTGCTGCCGACCGCTGCTGC	TIR library PelBss
123	CAGTCCTAGGTACAGTGCTAGCTACTAGAGATTAAAGAGGAGAAANNNNNNATGAARAARAGAGGGGCGTTTTTAGGGCT	TIR library YebFss
124	CCAAATTCCAGAAAAGAGACGCTTTCGAGCGTCTTTTTTCGTTTTGGTCCACCGTATTCATCAGCACTGTCCTGCTCCTT	*galK* integration 1./2. PCR
125	AATTCATTAAAGGTTGACAATTAATCATCGGCATAGTATATCGGCATAGTATAATACGACAAGGTGAGGAACTAAACCCAGGAGGCAGATCATGAGGCTTAAAGAAAAAACACAATCTCTGTTTG	*galK* integration 1. PCR
126	CGCCCAGCCAGTGGCGTCATCTCAATTCGCAGAAAGATTAAGCGCAACGCATAAAACGAAAGGCCCAGTCTTTCGACTGAGCCTTTCGTTTTATAATTCATTAAAGGTTGACAATTAATCATCGGCATAG	*galK* integration 2. PCR
127	CAGTACGAAAATTGCTTTCATATATAGTTTCTCCTCTTTAATCTCTAGTAGCTAGCACTGTACCTAGGACTGAGCTAGCCGTCAAGCGCAACGCGGACCAAAACGAAAAAAGACGCTCGAAAGCGTCTCTTTTCTGGAATTTGG	*galK* PrhaBAD to J23100 exchange
128	GTTTGCGCGCAGTCAGCGATATCCATTTTCGCGAATCCGGAGTGTAAGAAGAATTCTGTAAACCATCACAAGGAGCAGGACAGTGCTGAACGAAACTCCCGCACTG	*galK* knock-out
129	CTGGCGATGCTGTCGGAATGGACGATATCCCGCAAGAGGCCCGGCAGTACTTAAACAAAATTATTTGTAGAGGC	Truncating *tetA* while integrating Marionette collection promoter as C1
130	CCAAATTCCAGAAAAGAGACGCTTTCGAGCGTCTTTTTTCGTTTTGGTCCGCTTAACGATCGTTGGCTG	Integrate Marionette C1 without regulator/truncate *tetA*
131	CCAAATTCCAGAAAAGAGACGCTTTCGAGCGTCTTTTTTCGTTTTGGTCCCCAATTAGGATCCTTA	Integrate Marionette C1 with regulator/truncate *tetA*
132	TCTGATGAGTCCGTGAGGACGAAACAGCCTCTACAAATAATTTTGTTTAATACTAGAGATTAAAGAGGAGAAA	TIR transfer into strain with Marionette C1 / 3' *tetA*
133	GATGCTGTCGGAATGGACGATATCCCGCAAGAGGCCCGGCAGTACCATTAGAAAACCTCCTTAGCATGATTAAGATGTTTCAGTACGAAA	C1-BCD-3'*tetA* truncation
134	ATAGTGACTGGCGATGCTGTCGGAATGGACGATATCCCGCAAGAGGCCCGGCAGTACTTATCATTTGTAGAGCTCATCCATGCCATGTGTAATCCCAGCAGCAGT	C1-C2-gadget-*gfp*-3'*tetA* truncation
135	ATTTTCGTACTGAAACATCTTAATCATGCTAAGGAGGTTTTCTAATGGTTTCCAAGGGCGAGGAGGA	mCherry integration

Number	Sequence	Description
136	CTAAGGATTTTTTTATCTGGACAATTGTCTCAGGTCGAGGTGG CCCGGCTTATTTGTACAGCTCATCCA	mCherry integration
137	CTAAGGATTTTTTTATCTGGACAATTGTCTCAGGTCGAGGTGG CCCGGCTTACTTGTACAGCTCGTCCA	RFP integration (reverse)
138	ATTTTCGTACTGAAACATCTTAATCATGCTAAGGAGGTTTTCTA ATGGTGAGCAAGGGCGAGGAGGA	RFP integration BCD
139	TGGAGCTGCAGAATGAAACCCAGTCAGCAACTTCTGACGCTTCT GGCGGTGTGAGCAAGGGCGAGGAGGA	RFP integration Ub
140	CTGTACTTCCAGAGCAAAGGAGTGAGCAAGGGCGAGGAGGA	RFP integration TEV
141	CTGTACTTCCAGTTTCGTAGCAAAGGAGAAGAACTTGTCACTGG AGTTGTGAGCAAGGGCGAGGAGGA	RFP integration PROTi
142	CGACCGCTGCTGCTGGTCTGCTGCTCCTCGCTGCCCAGCCGGCG ATGGCCGTGAGCAAGGGCGAGGAGGA	RFP integration PelBss
143	ATTTTCGTACTGAAACATCTTAATCATGCTAAGGAGGTTTTCTA ATGGCTCAGGTCCAAC	Integration of Nanobody
144	CTAAGGATTTTTTTATCTGGACAATTGTCTCAGGTCGAGGTGG CCCGGCTCAGTGGTGGTGGTGGTGAT	Integration of Nanobody
145	ATTTTCGTACTGAAACATCTTAATCATGCTAAGGAGGTTTTCTA ATGACGGTCTGCGCAAAAAAACA	Integration of *crtE* / *crtEBIY*
146	CTAAGGATTTTTTTATCTGGACAATTGTCTCAGGTCGAGGTGG CCCGGCTTAACGATGAGTCGTCATAA	Integration of *crtEBIY*
147	CTGGCGATGCTGTCGGAATGGACGATATCCCGCAAGAGGCCCGG CAGTACTTATTAACTGACGGCAGCGAG	Integration of *crtE* / truncating *tetA*
148	ATTTTATTCAGGCCTGGTTTG	Integration of *crtB*
149	CTGGCGATGCTGTCGGAATGGACGATATCCCGCAAGAGGCCCGG CAGTACTTATATCAGATCCTCCAGCATC	Integration of *crtI* / truncating *tetA*
150	GGGCTCATCCTCCCCGCCCT	Integration of *crtI*
151	TCGGCTCGGCAAAAGCGACA	Integration of *crtY*
152	ATTTTCGTACTGAAACATCTTAATCATGCTAAGGAGGTTTTCTA ATGGATTCGCAACGCAATCTTTT	Integration of *yidC-gfp*-5'*tetA* in BCD-3'*tetA* strains
153	AAAAAGCGCTCGGAATTTAATTATTTTAAGAGATAAAACCGTCT GCGGAACTCGGTACCAAATTCCAGAA	Intergenic integration *yagF*
154	TTGTTTAAATTAATAAATTTCAAAAACAACAAAGCCGTCTGCGG GAAATAAGAAATCATCCTTAGCGAAA	Intergenic integration *yagF*
155	AGGCGGGTGAGAGCAATATTGGTATAATTTTTCAGCAATAAGAC CCTCGGTACCAAATTC	Intergenic integration *hupA*
156	GAAGACTCCAGGTACGACAAATCAGGCGTTAAATCACGTTTTCT TAGAAATCATCCTTAG	Intergenic integration *hupA*
157	ATCGCAACTTTGATCGAATTTCATCAGTTTTTCACCCGTAATAT ACTCGGTACCAAATTC	Intergenic integration *wbbL*
158	ATTAAAGTATAAATAGCTTATCCATGCTTATATGCTTACGGCTT TAGAAATCATCCTTAG	Intergenic integration *wbbL*
159	AAAAAATGGTAGATGAATACCAACAAAACTCTGGGCAGTAATAA ACTCGGTACCAAATTC	Intergenic integration *ykgH*
160	ATTGTCACTTCAGCTTTATACAGGCACTCTCTATCAGAATGTTG TAGAAATCATCCTTAG	Intergenic integration *ykgH*

Number	Sequence	Description
161	TTCCAGCAAAAATTCTTCCCGATCGTCATTACCAGCTGACGTGATAGAAATCATCCTTAG	Intergenic integration *tam*
162	GGCATTTCCGCGTCTGTTTATTGTTGCCCGGCGTATGGAGTAAATCTCGGTACCAAATTC	Intergenic integration *tam*
163	AGCTGGCACGACAGGTTTCCCGACTGGAAAGCGGGCAGTGAGCGCAGAAATCATCCTTAG	Intergenic integration *lacZ*
164	AGCCTGGGGTGCCTAATGAGTGAGCTAACTCACATTAATTGCGTTCTCGGTACCAAATTC	Intergenic integration *lacZ*
165	TTTTAACAGCCAATGATGGTTCTTAGCGCCGATTTTTAGCAGACTAGAAATCATCCTTAG	Intergenic integration *rsmG-atpI*
166	AGCAAATAAAAATTTAATTTTTATCAAAAAAATCATAAAAAATTGACTCGGTACCAAATTC	Intergenic integration *rsmG-atpl*
167	GATAGTCAATATGTTCTGTTGAAGCAATTATACTGTATGCTCATACAGTAAGAAATCATCCTTAGCGAAA	Intergenic integration *recA*
168	CTGCGTATGCATTGCAGACCTTGTGGCAACAATTTCTACAAAACACTTGACTCGGTACCAAATTCCAGAA	Intergenic integration *recA*
173	TGCTGTAGCTGTGTACCGAAGACTGCACTTAAGTTGGCGCGTTAGAGAAATCATCCTTAG	Intergenic integration *ycbX*
174	CCGCAGGTTAATGTTGACAGCTTCAGCCTCGAACAGGCAGTCTAACTCGGTACCAAATTC	Intergenic integration *ycbX*
175	TAATGGGGTTACCTTCGCCGTCGATGTTCAAGAAAACACCCGATAACTTTAGAAATCATCCTTAGCGAAA	Intergenic integration *atoB*
176	GCGATGGTGATTGAACGGTTGAATTAATCAATAAAAACACCCGATAGCGCTCGGTACCAAATTCCAGAA	Intergenic integration *atoB*
177	TATTCAGTCAATTACAAACATTAATAACGAAGAGATGACAGAAAAATTTTAGAAATCATCCTTAGCGAAA	Intergenic integration *glmS-pstS*
178	CCGACGTTGACCAGCCGCGTAACCTGGCAAAATCGGTTACGGTTGAGTAATAAATGGATCTCGGTACCAAATTCCAGAA	Intergenic integration *glmS-pstS*
179	AAAGTATAGGAACTTCAGAGCGCTTTTGAAGCTCACGCTGCCGCAAGCACCTCGGTACCAAATTCCAGAA	Integration in KEIO collection strains
180	AGAATAGGAACTTCGGAATAGGAACTTCAAGATCCCCTTATTAGAAGAACAGAAATCATCCTTAGCGAAA	Integration in KEIO collection strains
Cloning oligonucleotides		
201	ATACGGTTAUGCTGAGGTCGCCTCAGC	J23100-BCD-*gfp-tetA* in pSEVA27
202	ATAACCGTAUTTACGCTAGCAGCACGCCATAG	J23100-BCD-*gfp-tetA* in pSEVA27
203	AGTCCTAGGUACAGTGCTAGCTACTAGAGATTAAAGAGGAGAAA	J23100-BCD-*gfp-tetA* in pSEVA27
204	ACCTAGGACUGAGCTAGCCGTCAAGCGCAACGCCAAATAAAACGAAAG	J23100-BCD-*gfp-tetA* in pSEVA27
205	ACTTCCAGTUTCGTAGCAAAGGAGAAGAACTTGTCACTGGAGTTAGCAAAGGAGAAGAACTTTT	Introduction of PROTi gadget
206	AACTGGAAGUACAGRTTYTCCATNNNNNNTTTCTCCTCTTTAATCTCTA	Introduction of PROTi gadget

Number	Sequence	Description
207	AGTTCTTCUCCTTTGCTCTGGAAGTACAGRTTYTCCATNNNNNNTTTCTCCTCTTTAATCTCTA	Introduction of TEV gadget
208	AGAAGAACUTTTCACTGGAGTTGTG	Introduction of TEV gadget, Ubiquitin gadget
209	AGTTCTTCUCCTTTGCTACCGCCAGAAGCGTCAGAAG	Introduction of Ubiquitin gadget
210	ACTAGAGATUAAAGAGGAGAAANNNNNNATGCARATHTTCGTGAAAACACTG	Introduction of Ubiquitin gadget
211	AATCTCTAGUAGCTAGCACTGTACCTAGGA	Introduction of Ubiquitin gadget
212	AACAGCCCUAAAAACGCCCCTCTYTTYTTCATNNNNNNTTTCTCCTCTTTAATCTCTA	cloning of sfGFP on YebF/ PelB plasmids, YebF gadget
213	AGGGCTGTUGTTGGTTTCTGCCTGCGCATCAGTTTTCGCTCGTAAAGGCGAAGAGCTGTT	cloning of sfGFP on YebF/ PelB plasmids, YebF gadget
214	ACCGCTGCUGCTGGTCTGCTGCTCCTCGCTGCCCAGCCGGCGATGGCCCGTAAAGGCGAAGAGCTGTT	cloning of sfGFP on YebF/ PelB plasmids, PelB gadget
215	AGCAGCGGUCGGCAGCAGRTAYTTCATNNNNNNTTTCTCCTCTTTAATCTCTA	cloning of sfGFP on YebF/ PelB plasmids, PelB gadget
216	ATGAAAAAATUATTTGCTTATTAATCATCCGGCTC	cloning of sfGFP on YebF/ PelB plasmids
217	AAATTTTTTCAUCATTTGTACAGTTCATCCATACCAT	cloning of sfGFP on YebF/ PelB plasmids
218	AGATCGATCUCGATCCCGCGAAAT	*tetA* homology removal on PT7/PTrc plasmids
219	AGATCGATCUCGATCCGACACCATCGAATGGCG	*tetA* homology removal on PT7/PTrc plasmids
220	AGGGGCGUTTTTAGGGCTG	YebF TIR on pSEVA27 reverse primer
221	ACGCCCCUCTCTTTTTCATAGCCCCTTTCTCCTCTTTAAT	YebF TIR high on pSEVA27
222	ACGCCCCUCTCTTTTTCATCCAGTCTTTCTCCTCTTTAAT	YebF TIR medium on pSEVA27
223	ACGCCCCUCTTTTCTTCATCGAGGGTTTCTCCTCTTTAAT	YebF TIR low on pSEVA27
224	AGTTTTCGCUGCGAATAACGAAACGAGCAA	cloning of full YebF protein: *yebF*
225	AACAGCTCTUCGCCTTTACGGCGGCGTTGGTACTC	cloning of full YebF protein: *yebF*
226	AAGAGCTGTUCACTGGTGTC	cloning of full YebF protein: Backbone
227	AGCGAAAACUGATGCGCAGG	cloning of full YebF protein: Backbone
228	AATCTCTAGUAGCTAGCACTGTACCTAGGA	pSEVA27-5'*tetA* backbone cloning

Number	Sequence	Description
229	AACCAATTATUGAAGGCCGCT	pSEVA27-5'*tetA* backbone cloning
230	ACTAGAGATUATAAGGAGATATACCTATGACGGTCTG	*crtEBIY* pathway on pSEVA27-5'*tetA*
231	AATAATTGGTUAACGATGAGTCGTCAT	*crtEBIY* pathway on pSEVA27-5'*tetA*
232	ACTAGAGATUGGGCTCATCCTCCCCGCCCT	*crtIY* on pSEVA27-5'*tetA*
233	AATAATTGGTUATTAGAGCGGGCGCTG	*crtB* on pSEVA27-5'*tetA*
234	ACTAGAGATUATTTTATTCAGGCCTGGTTTG	*crtB* on pSEVA27-5'*tetA*
235	AGTGAAGGTAUCCTCAGCCGCGCGCGC	Backbone amplification for pSEVA27-*yidC-gfp*-5'*tetA*
236	AGTTCGGTUACGAGATCCATTTGTC	Backbone amplification for pSEVA27-*yidC-gfp*-5'*tetA*
237	AGGCCGCUAACGCGGCCTTTTTTTGTTTCTGGTCTCCCAAAAATTTATTTGCTTATTA	Backbone amplification for pSEVA27-*yidC-gfp*-5'*tetA*
238	AACCGAACUTGAGAACAACCAGATA	Backbone amplification for pSEVA27-*yidC-gfp*-5'*tetA*
239	ATACCTTCACUGCCCGCTTTCCAGTC	YidC-gfp amplification for pSEVA27-*yidC-gfp*-5'*tetA*
240	AGCGGCCUTCAATAATTGGTCATTTGTAGAGCTCATCCA	YidC-gfp amplification for pSEVA27-*yidC-gfp*-5'*tetA*
241	ATTTGCCAAUTAGGATCCTTA	Recloning of Marionette plasmids for C1 integration
242	AGCTCCAAUGGCGGCGCGCCATCGAAT	Recloning of Marionette plasmids for C1 integration
243	ATTGGCAAAUAAAACGAAAGGCTCA	Recloning of Marionette plasmids for C1 integration
244	ATACTAGAGATUAAAGAGGAGAAA	Recloning of Marionette plasmids for C1 integration
245	AATCTCTAGTAUTAAACAAAATTATTTGTAGA	Recloning of Marionette plasmids for C1 integration
246	ATTGGAGCUTAACGATCGTTGGCTG	Recloning of Marionette plasmids for C1 integration
Sequencing oligonucleotides		
301	TTCATGTGATCCGGATAACGGG	*gfp* reverse
302	TTCCGGCATGGCGGACTTGA	*sfgfp* reverse
303	GGCTTACCATGTCGCGCTGATCA	Landing pad upstream (*glmS)*
304	GGTGATGTCGGCGATATAGG	*tetA* reverse
305	GCTCAGTCCTAGGTACAGTGCT	J23100 forward
306	AATGTCTCCTGGGAGGATTC	Landing pad downstream (*pstS*)

Number	Sequence	Description
307	CGCTATCATGCCATACCG	PlacI reverse

Supplementary Table 4.

Supplementary Table 4. Sequences of recombineering sites

Site	Middle gadget		Sequence (5' – 3')	Comments
**RS1**			CCAAATTCCAGAAAAGAGACGCTTTCGAGCGTCTTTTTTCGTTTTGGTCC	Use for integration of control bricks / controlled cargo brick
**RS2-control**	BCD		CAGTACGAAAATTGCTTTCATATATAGTTTCTCCTCTTTAATCTCTAGTA	Use for integration of control bricks
**RS2-cargo**	BCD		ATTTTCGTACTGAAACATCTTAATCATGCTAAGGAGGTTTTCTAATG	Use for integration of SEGA cargo bricks
	Ubiquitin		TGGAGCTGCAGAATGAAACCCAGTCAGCAACTTCTGACGCTTCTGGCGGT	
	TEV*	C2: low	TACTAGAGATTAAAGAGGAGAAACGGGGGATGGAGAACCTGTACTTCCAG	
		C2: med	TACTAGAGATTAAAGAGGAGAAAAACTACATGGAAAACCTGTACTTCCAG	
		C2: high	TACTAGAGATTAAAGAGGAGAAAATATCGATGGAGAACCTGTACTTCCAG	
	PROTi		CTGTACTTCCAGTTTCGTAGCAAAGGAGAAGAACTTGTCACTGGAGTT	
	PelB		CGACCGCTGCTGCTGGTCTGCTGCTCCTCGCTGCCCAGCCGGCGATGGCC	
	YebF		ATCAGGTGAGCGTCGATTGCAAAGCCGGCATGGCGGAGTACCAACGCCGC	
**RS3**			CTAAGGATTTTTTTTATCTGGACAATTGTCTCAGGTCGAGGTGGCCCGGC	Use for integration of cargo bricks / controlled cargo bricks
**RS3-trunc**			CTGGCGATGCTGTCGGAATGGACGATATCCCGCAAGAGGCCCGGCAGTAC	Use for truncation of *tetA*
**RS3-recon**			CGCTAGCAGCACGCCATAGTGACTGGCGATGCTGTCGGAATGGACGATATCCCGCAAGAGGCCCGGCAGTAC	Use for reconstitution of *tetA*

*In the case of the TEV-mGadget it is necessary to use homology sequences that are specific for each C2 site in order to create a scarless integration with the possibility to create a custom N-terminus on demand.

Supplementary Table 5.

Supplementary Table 5. Optimized sequences of the translational control element (C2)

C2 / middle gadget	Low translation strength	Medium translation strength	High translation strength
**BCD**	GGGGGG**ATG**AAGGCT	GAGCTA**ATG**AAGGCT	CTATAT**ATG**AAAGCA
**Ubiquitin**	GGGGGG**ATG**CAAATT	GGCTGG**ATG**CAAATA	CATTGT**ATG**CAGATA
**TEV site**	CGGGGG**ATG**GAGAAC	AACTAC**ATG**GAAAAC	ATATCG**ATG**GAGAAC
**PROTi**	CGGGGG**ATG**GAGAAC	AACTAC**ATG**GAAAAC	ATATCG**ATG**GAGAAC
**PelBss**	CGGACG**ATG**AAGTAC	TCAATG**ATG**AAGTAC	TTAATT**ATG**AAATAT
**YebF**	CCCTCG**ATG**AAGAAA	GACTGG**ATG**AAAAAG	GGGGCT**ATG**AAAAAG

Supplementary Table 6.

Supplementary Table 6. Homology regions for the integration into different genomic loci

Locus	Distance to ori [bp]	Integration Start (5’)	Homology upstream	Homology downstream
rsmG-atpI	2686	3923027	AGCAAATAAAATTTAATTTTTATCAAAAAAATCATAAAAAATTGA	TTTTAACAGCCAATGATGGTTCTTAGCGCCGATTTTTAGCAGAC
glmS-pstS	13904	3911719	CCGACGTTGACCAGCCGCGTAACCTGGCAAAATCGGTTACGGTTGAGTAATAAATGGAT	TATTCAGTCAATTACAAACATTAATAACGAAGAGATGACAGAAAAATTTT
hupA	274538	4200098	AGGCGGGTGAGAGCAATATTGGTATAATTTTTCAGCAATAAGACC	GAAGACTCCAGGTACGACAAATCAGGCGTTAAATCACGTTTTCT
yagF	999110	285198	AAAAAGCGCTCGGAATTTAATTATTTTAAGAGATAAAACCGTCTGCGGAA	TTGTTTAAATTAATAAATTTCAAAAACAACAAAGCCGTCTGCGGGAAATA
ykgH	1040605	324691	AAAAAATGGTAGATGAATACCAACAAAACTCTGGGCAGTAATAAA	ATTGTCACTTCAGCTTTATACAGGCACTCTCTATCAGAATGTTG
lacZ	1079140	366423	AGCCTGGGGTGCCTAATGAGTGAGCTAACTCACATTAATTGCGTT	AGCTGGCACGACAGGTTTCCCGACTGGAAAGCGGGCAGTGAGCGC
recA	1103101	2823848	CTGCGTATGCATTGCAGACCTTGTGGCAACAATTTCTACAAAACACTTGA	GATAGTCAATATGTTCTGTTGAAGCAATTATACTGTATGCTCATACAGTA
atoB	1598340	2327315	GCGATGGTGATTGAACGGTTGAATTAATCAATAAAAACACCCGATAGCG	TAATGGGGTTACCTTCGCCGTCGATGTTCAAGAAAACACCCGATAACTTT
ycbX	1722400	1006494	CCGCAGGTTAATGTTGACAGCTTCAGCCTCGAACAGGCAGTCTAA	TGCTGTAGCTGTGTACCGAAGACTGCACTTAAGTTGGCGCGTTAG
wbbL	1822799	2101391	ATCGCAACTTTGATCGAATTTCATCAGTTTTTCACCCGTAATATA	ATTAAAGTATAAATAGCTTATCCATGCTTATATGCTTACGGCTT
tam	2318397	1608106	GGCATTTCCGCGTCTGTTTATTGTTGCCCGGCGTATGGAGTAAAT	TTCCAGCAAAAATTCTTCCCGATCGTCATTACCAGCTGACGTGA
KEIO*		46*	AAAGTATAGGAACTTCAGAGCGCTTTTGAAGCTCACGCTGCCGCAAGCAC	AGAATAGGAACTTCGGAATAGGAACTTCAAGATCCCCTTATTAGAAGAAC

*The same homology sequences were used for all integrations into KEIO collection strains. The position of the integration is given relative to the sequence of the KEIO knock-out cassette. For details on the construction of the KEIO strains see Baba *et al.* (2006)[5].

References

1. Rennig, M. *et al.* TARSyn: Tunable Antibiotic Resistance Devices Enabling Bacterial Synthetic Evolution and Protein Production. *ACS Synth Biol* **7**, 432–442 (2018).
2. Meyer, A. J., Segall-Shapiro, T. H., Glassey, E., Zhang, J. & Voigt, C. A. Escherichia coli "Marionette" strains with 12 highly optimized small-molecule sensors. *Nature Chemical Biology* **15**, 196–204 (2019).
3. Virtanen, P. *et al.* SciPy 1.0: fundamental algorithms for scientific computing in Python. *Nat Methods* **17**, 261–272 (2020).
4. Mundhada, H., Schneider, K., Christensen, H. B. & Nielsen, A. T. Engineering of high yield production of L-serine in Escherichia coli. *Biotechnology and Bioengineering* **113**, 807–816 (2016).
5. Baba, T. *et al.* Construction of Escherichia coli K-12 in-frame, single-gene knockout mutants: the Keio collection. *Molecular Systems Biology* **2**, 2006.0008 (2006).
6. Bonde, M. T. *et al.* Predictable tuning of protein expression in bacteria. *Nat Methods* **13**, 233–236 (2016).
7. Datta, S., Costantino, N. & Court, D. L. A set of recombineering plasmids for gram-negative bacteria. *Gene* **379**, 109–115 (2006).
8. Mirzadeh, K. *et al.* Enhanced Protein Production in Escherichia coli by Optimization of Cloning Scars at the Vector–Coding Sequence Junction. *ACS Synth. Biol.* **4**, 959–965 (2015).
9. Martínez, V. *et al.* CRISPR/Cas9-based genome editing for simultaneous interference with gene expression and protein stability. *Nucleic Acids Research* **45**, e171–e171 (2017).
10. Cavaleiro, A. M., Kim, S. H., Seppälä, S., Nielsen, M. T. & Nørholm, M. H. H. Accurate DNA Assembly and Genome Engineering with Optimized Uracil Excision Cloning. *ACS Synth. Biol.* **4**, 1042–1046 (2015).
11. Daley, D. O. *et al.* Global Topology Analysis of the Escherichia coli Inner Membrane Proteome. *Science* **308**, 1321–1323 (2005).

12. Kim, S. H., Cavaleiro, A. M., Rennig, M. & Nørholm, M. H. H. SEVA Linkers: A Versatile and Automatable DNA Backbone Exchange Standard for Synthetic Biology. https://pubs.acs.org/doi/pdf/10.1021/acssynbio.5b00257 (2016) doi:10.1021/acssynbio.5b00257.
