# Supplementary material for: A standardized genome architecture for bacterial synthetic biology (SEGA)
Source: Nat Commun. 2021 Oct 7;12:5876. doi: 10.1038/s41467-021-26155-5 (PMC8497626; doi:10.1038/s41467-021-26155-5)
Supplement: Supplementary file 2 — Reporting Summary [file 41467_2021_26155_MOESM2_ESM.pdf]

nature portfolio

Corresponding author(s): Maja Rennig, Morten H.H. Nørholm

Last updated by author(s): Sep 7, 2021

Reporting Summary

Nature Portfolio wishes to improve the reproducibility of the work that we publish. This form provides structure for consistency and transparency in reporting. For further information on Nature Portfolio policies, see our Editorial Policies and the Editorial Policy Checklist.

Statistics

For all statistical analyses, confirm that the following items are present in the figure legend, table legend, main text, or Methods section.

n/a	Confirmed	
☐	☒	The exact sample size (*n*) for each experimental group/condition, given as a discrete number and unit of measurement
☐	☒	A statement on whether measurements were taken from distinct samples or whether the same sample was measured repeatedly
☒	☐	The statistical test(s) used AND whether they are one- or two-sided<br>*Only common tests should be described solely by name; describe more complex techniques in the Methods section.*
☒	☐	A description of all covariates tested
☒	☐	A description of any assumptions or corrections, such as tests of normality and adjustment for multiple comparisons
☐	☒	A full description of the statistical parameters including central tendency (e.g. means) or other basic estimates (e.g. regression coefficient) AND variation (e.g. standard deviation) or associated estimates of uncertainty (e.g. confidence intervals)
☒	☐	For null hypothesis testing, the test statistic (e.g. *F*, *t*, *r*) with confidence intervals, effect sizes, degrees of freedom and *P* value noted<br>*Give P values as exact values whenever suitable.*
☒	☐	For Bayesian analysis, information on the choice of priors and Markov chain Monte Carlo settings
☒	☐	For hierarchical and complex designs, identification of the appropriate level for tests and full reporting of outcomes
☒	☐	Estimates of effect sizes (e.g. Cohen's *d*, Pearson's *r*), indicating how they were calculated

Our web collection on statistics for biologists contains articles on many of the points above.

Software and code

Policy information about availability of computer code

Data collection	FACS sorting: Sony Cell Sorter Software (v2.1.5), Flow cytometry: MACSQuantify (v2.13.1), Microplate reader: BioTek Gen5 (v 3.08), iBright gel imager software (v1.6.0)
Data analysis	Data processing: Microsoft Excel 2016, Graphics and linear regression: GraphPad Prism (v9.1.0), FACS and flow cytometry: FlowJo (v 10.6.1)

For manuscripts utilizing custom algorithms or software that are central to the research but not yet described in published literature, software must be made available to editors and reviewers. We strongly encourage code deposition in a community repository (e.g. GitHub). See the Nature Portfolio guidelines for submitting code & software for further information.

Data

Policy information about availability of data

All manuscripts must include a data availability statement. This statement should provide the following information, where applicable:

- Accession codes, unique identifiers, or web links for publicly available datasets
- A description of any restrictions on data availability
- For clinical datasets or third party data, please ensure that the statement adheres to our policy

FACS data for Figure 3 and Supplementary Figures 6 - 10 were deposited at FlowRepository (http://flowrepository.org) under accession codes FR-FCM-Z43R and FR-FCM-Z445. DNA sequences are provided with the supplementary material of this paper. Relevant strains of the SEGA collection are deposited at the Belgian Co-ordinated Collections of Microorganisms (https://bccm.belspo.be) and the repository IDs can be found on www.sega-genomes.com/straincollection. The data for bar or dot graphs generated in this study are provided in the Source Data file.

Field-specific reporting

Please select the one below that is the best fit for your research. If you are not sure, read the appropriate sections before making your selection.

☒ Life sciences ☐ Behavioural & social sciences ☐ Ecological, evolutionary & environmental sciences

For a reference copy of the document with all sections, see nature.com/documents/nr-reporting-summary-flat.pdf

Life sciences study design

All studies must disclose on these points even when the disclosure is negative.

Sample size	All experiments were performed as biological triplicates unless stated otherwise. No statistical methods were applied to determine the sample size. The results were reproducible across replicates and thus the sample size was deemed sufficient. Triplicates were chosen as the sample size since it is considered a valid analytical approach in the field.
Data exclusions	Figure 5: Two strains with integrations of the SEGA landing pad in particular genome locations were excluded after mutations in the tetA selection marker were found. The two strains were excluded from all analyses. Figure 4: Two replicates of the uninduced sample of PTrc-low-BCD-YidC-gfp were excluded after mutations in the gfp coding sequence were found. These exclusion criteria are pre-established since mutations in coding-regions can alter protein levels and therefore the results obtained for these samples are not reliable.
Replication	All experiments were performed as biological triplicates, unless stated otherwise. All attempts at replication were successful.
Randomization	No randomization was performed as it is not relevant for the presented study. In the field, randomization is not considered a necessary analytical approach for the presented experiments.
Blinding	No blinding was performed as it is not relevant for the presented study. In the field, blinding is not considered a necessary analytical approach for the presented experiments.

Reporting for specific materials, systems and methods

We require information from authors about some types of materials, experimental systems and methods used in many studies. Here, indicate whether each material, system or method listed is relevant to your study. If you are not sure if a list item applies to your research, read the appropriate section before selecting a response.

Materials & experimental systems

n/a	Involved in the study
☒	☐ Antibodies
☒	☐ Eukaryotic cell lines
☒	☐ Palaeontology and archaeology
☒	☐ Animals and other organisms
☒	☐ Human research participants
☒	☐ Clinical data
☒	☐ Dual use research of concern

Methods

n/a	Involved in the study
☒	☐ ChIP-seq
☐	☒ Flow cytometry
☒	☐ MRI-based neuroimaging

Flow Cytometry

Plots

Confirm that:

☒ The axis labels state the marker and fluorochrome used (e.g. CD4-FITC).

☒ The axis scales are clearly visible. Include numbers along axes only for bottom left plot of group (a 'group' is an analysis of identical markers).

☒ All plots are contour plots with outliers or pseudocolor plots.

☒ A numerical value for number of cells or percentage (with statistics) is provided.

Methodology

Sample preparation	Bacterial cells in culture medium or diluted in PBS
Instrument	(1) Sony SH800S, (2) MACSQuant VYB

Software	Collection: (1) Sony Cell Sorter Software (v2.1.5), (2) MACSQuantify (v2.13.1), Analysis: FlowJo (v10.6.1)
Cell population abundance	Typically 10,000 cells were analyzed for each sample
Gating strategy	The experimental threshold was set to 3000 (MACSQuant) or 1.5% (Sony) respectively. No further initial gating strategy was applied and therefore no cells excluded from the experiment. The gating to select low, medium and high expressing variants is presented in Figure 3C and Supplementary Figure 6.

☒ Tick this box to confirm that a figure exemplifying the gating strategy is provided in the Supplementary Information.
