# Supplementary material for: A standardized genome architecture for bacterial synthetic biology (SEGA)
Source: Nat Commun. 2021 Oct 7;12:5876. doi: 10.1038/s41467-021-26155-5 (PMC8497626; doi:10.1038/s41467-021-26155-5)
Supplement: Supplementary file 3 — Description of Additional Supplementary Files [file 41467_2021_26155_MOESM3_ESM.pdf]

Title: Supplementary Data 1
Description: SEGA strain table
